# Three-dimensional exponential mixing and ideal kinematic dynamo with randomized ABC flows

**DOI:** 10.1007/s10884-026-10483-5

**Published:** 2026-02-06

**Authors:** Michele Coti Zelati, Víctor Navarro-Fernández

**Affiliations:** Department of Mathematics, Imperial College London, LONDON, SW7 2AZ UK

**Keywords:** ABC flows, Exponential mixing, Ideal kinematic dynamo, 35Q49, 37A25, 76F25, 76W05

## Abstract

In this work we consider the Lagrangian properties of a random version of the Arnold–Beltrami–Childress (ABC) flows in a three-dimensional periodic box. We prove that the associated flow map possesses a positive top Lyapunov exponent and its associated one-point, two-point and projective Markov chains are geometrically ergodic. For a passive scalar, it follows that such a velocity is a space-time smooth exponentially mixing field, uniformly in the diffusivity coefficient. For a passive vector, it provides an example of a universal ideal (i.e. non-diffusive) kinematic dynamo.

## Introduction

The study of turbulent and chaotic dynamical systems holds significant interest in both mathematics and physics due to its ability to uncover the inherent unpredictability and complexity found in various natural phenomena, such as weather patterns and fluid behavior. Serving as a quintessential example of a simple flow with intricate dynamics, this paper examines the *Arnold–Beltrami–Childress* (ABC) flows. These flows are characterized by a set of parameters $\textsf{A},\textsf{B},\textsf{C}\in \mathbb {R}$ and are defined on the three-dimensional torus $\mathbb {T}^3=\mathbb {R}^3/(2\pi \mathbb {Z})^3$ by the vector field

1.1 $$\begin{aligned} u(\boldsymbol{x}) = \begin{pmatrix} \textsf{A}\sin z + \textsf{C}\cos y \\ \textsf{B}\sin x+ \textsf{A}\cos z \\ \textsf{C}\sin y + \textsf{B}\cos x \end{pmatrix} \in \mathbb {R}^3, \end{aligned}$$

where we write $\boldsymbol{x}= (x,y,z) \equiv (x\mod 2\pi , y\mod 2\pi , z\mod 2\pi )\in \mathbb {T}^3$.

This class of vector fields possesses numerous intriguing properties that render them relevant for various applications in mathematical physics. Firstly, they are divergence-free, meaning $\nabla \cdot u = 0$. Secondly, they satisfy the so-called *Beltrami* property, which means that there holds the identity $u=\nabla \times u$ for all $\textsf{A},\textsf{B},\textsf{C}\in \mathbb {R}$. In particular, they define a stationary solution to the 3*d* Euler equations for an inviscid fluid. Since the work of Arnold [4], ABC flows have been considered as extraordinary examples of simple flows with a complicated topology and Lagrangian structure. If one of the parameters $\textsf{A}$, $\textsf{B}$ or $\textsf{C}$ is zero, the flow is integrable. However, if all three parameters are nonzero, the behavior of the streamlines becomes complex and unpredictable, indicative of chaos [24, 29].

Another significant context in which the ABC flows arise is magnetohydrodynamics, particularly when studying how the movement of a magnetic fluid can alter the intensity of the magnetic field itself. This is related to the *dynamo effect*, which is of particular interest in fields such as geophysics and astrophysics for explaining phenomena like stellar magnetism [14]. Childress [15] proposed the ABC flows as potential candidate vector fields that could cause the magnetic field’s intensity to grow rapidly.

In this paper, we will study how the Lagrangian structure of a randomized version of (1.1) exhibits chaotic behavior. Specifically, this has significant consequences from the point of view of mixing of passive scalars and ideal dynamo in the context of ideal magnetohydrodynamics.

### Mixing of passive scalars

The mixing properties of a divergence-free vector field $u:[0,\infty )\times \mathbb {T}^3\rightarrow \mathbb {R}^3$ are typically understood in terms of its ability to transfer the energy of a passive tracer from large to small spatial scales. Let $\kappa \in [0,1]$ be a diffusion parameter, and consider the Cauchy problem

1.2 $$\begin{aligned} {\left\{ \begin{array}{ll} \partial _t \rho + u\cdot \nabla \rho = \kappa \Delta \rho , \quad & \text {in }(0,\infty )\times \mathbb {T}^3,\\ \rho (0,\cdot ) = \rho _0, &  \text {in }\mathbb {T}^3. \end{array}\right. } \end{aligned}$$

The scalar function $\rho :[0,\infty )\times \mathbb {T}^3\rightarrow \mathbb {R}$ can represent, for instance, the concentration of some chemical in a solution, or the temperature of a gas in a room, starting at a suitable, mean-free initial datum $\rho _0$.

**Fig. 1:**
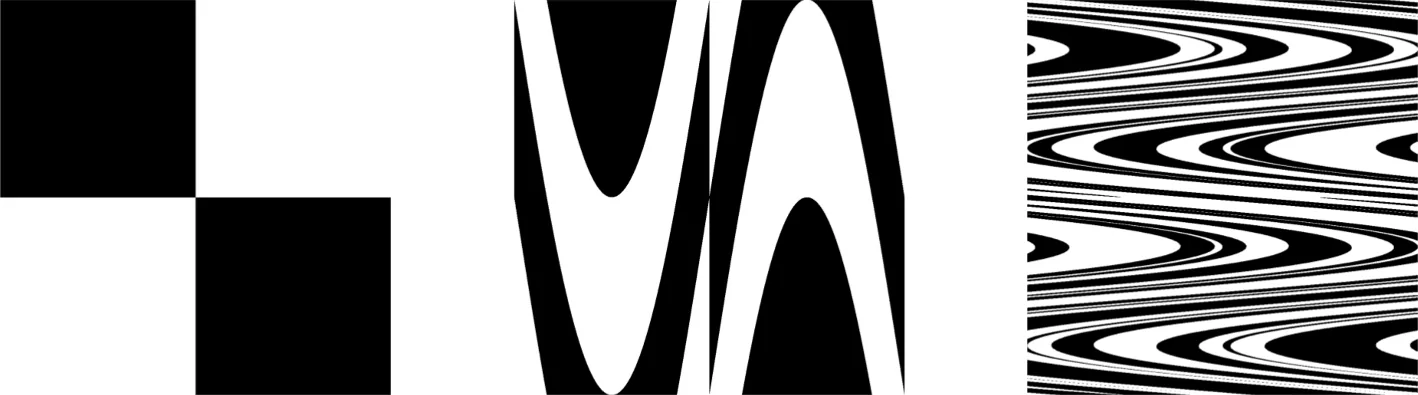
Mixing by alternating shear flows in $\mathbb {T}^2$

The effect of a divergence free-vector field can create complicated and chaotic structures in the evolution of the passive scalar, see Figure 1, where the filamentation and the decreasing typical length-scale that can be appreciated is symptomatic of mixing. One way to quantify mixing for (1.2) is via the so-called *functional mixing scale*, that involves the decay of negative Sobolev norms, defined through the Fourier transform $\widehat{\rho }$ by

1.3 $$\begin{aligned} \Vert \rho (t)\Vert _{\dot{H}^{-s}}^2 = \sum _{\boldsymbol{k}\in \mathbb {Z}^3_0} \frac{|\widehat{\rho }(t,\boldsymbol{k})|^2}{|\boldsymbol{k}|^{2s}}, \qquad s>0, \qquad \mathbb {Z}^3_0 = \mathbb {Z}^3\setminus \{(0,0,0)\}. \end{aligned}$$

The use of negative Sobolev norms is enlightening about the cascading mechanism to higher frequencies (i.e. small scales). The conservation of the $L^2$ norm implies that the sum of all modes $|\widehat{\rho }(t,\boldsymbol{k})|^2$ must be constant in time, therefore if the $H^{-s}$ norm of ρ(t) converges to zero as $t\rightarrow \infty$, it must occur that the mass concentrated in the lower modes ($|\boldsymbol{k}|$ small) is transported to the higher modes ($|\boldsymbol{k}|$ large).

This definition of mixing was first introduced in [36], and since then there have been a number of results and applications, some relevant examples of which are [1, 25, 35]. Other more geometrical definitions of mixing that under special circumstances might be equivalent to the one adopted here have been introduced to address these problems. For a review about the different ways of quantifying mixing, see [42].

It is well known that for sufficiently regular vector fields, mixing cannot be faster than exponential. This was proved for $u\in L^1_tW^{1,p}_{\boldsymbol{x}}$, $p\in (1,\infty ]$, in [22] (see also [30, 39]). Finding such lower bound for p=1 is related to Bressan’s conjecture [13] and remains open as of today.

In this work we are interested in almost sure exponentially fast mixing in the context of randomized vector fields, where $u=u(t,\boldsymbol{x},\omega )$ depends additionally on a random parameter ω. Some examples of almost-sure exponential mixers have been recently provided via vector fields that are solutions to stochastically forced Navier–Stokes equations [9, 10], and via alternating shear flows in $\mathbb {T}^2$ with random phases [11], or with random switching times [18]. We provide an examples of a smooth, bounded exponentially mixing field in this article (see Theorem 1).

The ideas presented in [11] in the inviscid case κ=0, have been very recently extended in [19] to the case of passive scalars that solve the advection-diffusion equation (1.2) with κ>0. This means that apart from the transport by the vector field, also the molecular diffusion of the passive scalar is taken into account. In [19], the decay of the mixing norm is proved to be *uniformly in the diffusion parameter*, so that the case without diffusion can be recovered as well. We will present our results using this strictly more general framework.

### Dynamos in passive vectors

The evolution of a three-dimensional magnetic field $B=B(t,\boldsymbol{x})$ in a homogeneous moving conductor is described by a combination of Maxwell’s equations and Ohm’s law, yielding the set of equations

1.4 $$\begin{aligned} {\left\{ \begin{array}{ll} \partial _t B + (u\cdot \nabla ) B - (B\cdot \nabla ) u = \kappa \Delta B ,\quad & \text {in }(0,\infty )\times \mathbb {T}^3, \\ \nabla \cdot B = 0, &  \text {in }(0,\infty )\times \mathbb {T}^3, \\ B(0,\cdot ) = B_0, &  \text {in }\mathbb {T}^3. \end{array}\right. } \end{aligned}$$

Here, *u* is the velocity field of the moving conductor, $\kappa \ge 0$ is the magnetic resistivity of the medium, and $B_0$ an assigned, mean-free initial condition.

Solutions of (1.4) can behave quite differently compared to those of (1.2). If *u* is Lipschitz, a standard stability estimate and the Poincaré inequality give that the $L^2$ norm of *B* cannot grow faster than exponential, due to the energy estimate

$$ \Vert B(t)\Vert _{L^2} \le \Vert B_0\Vert _{L^2} \textrm{e}^{(\Vert \nabla u\Vert _{L_{t,x}^\infty }-\kappa )t}. $$

The potential growth of the $L^2$ norm is due to the stretching term $(B\cdot \nabla )u$. Based on the conventions adopted in [6], we define different dynamo behaviors in terms of the asymptotic growth rate

1.5 $$\begin{aligned} \chi (\kappa ):= \sup _{B_0\in L^2} {\liminf _{t\rightarrow \infty }} \frac{1}{t} \log \Vert B(t)\Vert _{L^2}, \end{aligned}$$

uniformly with respect of initial data in $L^2$:

- *Ideal dynamo*: χ(0)>0. This involves only the non-resistive case, when we set κ=0 in (1.4), and postulate the existence of a velocity field that exhibits exponential growth of the magnetic field. We will provide an example below (see Theorem 2);
- *Fast dynamo*: $\chi _0:= \displaystyle \liminf _{\kappa \rightarrow 0}\chi (\kappa )>0$. Informally, the exponential growth rate of $\Vert B(t)\Vert _{L^2}$ becomes independent of κ in the perfectly conducting limit, and whether this happens is an outstanding open problem, formulated by Y.B. Zeldovich in 1957 in [48], and mentioned in the Arnold’s problem monograph [5, 1994-28] (see also [6, Chapter V] or [16]). Recently, an example of a vector field exhibiting fast dynamo in $\mathbb {R}^3$ was found in [21].
- *Slow dynamo*: $\chi _0:= \displaystyle {\limsup _{\kappa \rightarrow 0}}\chi (\kappa )\le 0$. In this case, the exponential growth is suppressed as $\kappa \rightarrow 0$. Evidence of this behavior, with $\chi (\kappa )\sim \kappa ^{1/3}$, is provided by the so-called Ponomarenko dynamo [27, 38].

Besides being interesting on its own from a mathematical perspective, this problem has relevant applications to astrophysics and geophysics, in particular to stellar magnetism [15, 33]. The intuitive idea behind a fast dynamo is the *Stretch–Twist–Fold* mechanism [16], which displays a discrete in time flow that *doubles* the density of magnetic lines on each iteration. ABC flows were proposed by Childress [15] as suitable candidates to be a fast dynamo in 3*d*, as their streamlines appear to be chaotic [4, 24, 47]. This claim is also supported by a strong numerical evidence [2, 12, 31].

### The Lagrangian picture and main results

The two problems described in Sections 1.1 and 1.2 share the same Lagrangian picture, in terms of the associated flow map

1.6 $$\begin{aligned} \textrm{d}\phi _t(\boldsymbol{x}) = u(t,\phi _t(\boldsymbol{x}))\textrm{d}t + \sqrt{2\kappa }\textrm{d}W_t, \quad \phi _0(\boldsymbol{x})=\boldsymbol{x}. \end{aligned}$$

where $W_t$ represents a standard Brownian motion with periodic boundary conditions in $\mathbb {T}^3$. Using the Feynman-Kac formula, solutions to (1.2) are given by

$$ \rho (t,\boldsymbol{x}) = \mathbb {E}_W\left[ (\phi _t)_\#\rho _0(\boldsymbol{x})\right] , $$

while for (1.4) we have

1.7 $$\begin{aligned} B(t,\boldsymbol{x}) = \mathbb {E}_W\left[ (\phi _t)_\#D_{\boldsymbol{x}} \phi _t B_0(\boldsymbol{x})\right] . \end{aligned}$$

The notation $(\phi _t)_\#$ typically denotes the push forward of a measure μ, i.e. $(\phi _t)_\#\mu (A) = \mu (\phi _t^{-1}(A))$ for any Borel set $A\subseteq \mathbb {T}^3$. When applied to measurable functions, it should be understood as the composition with the inverse of the flow map $(\phi _t)_\#f(\boldsymbol{x}) = f(\phi _t^{-1}(\boldsymbol{x}))$. In both cases above, the expectation is taken with respect to the noise associated to the Brownian motion, and it is therefore denoted by $\mathbb {E}_W$. Nonetheless, this may not be the only source of randomness, as *u* may contain some stochasticity itself. This is indeed the point of view we take here.

Let U>0 be a fixed real number, and consider two collections of random variables $(\textsf{A},\textsf{B},\textsf{C})\in [-U,U]^3\subset \mathbb {R}^3$, $(\alpha ,\beta ,\gamma )\in [0,2\pi )^3\subset \mathbb {R}^3$ which are independent and identically distributed (IID). These variables represent random amplitudes and random phases respectively that will be inserted in the ABC vector field (1.1). We define the probability space where the random variables take values as $(\Omega _0,\mathcal {F}_0,\mathbb {P}_0)$, with

$$ \Omega _0 = [-U,U]^3\times [0,2\pi )^3, \quad \mathcal {F}_0=\mathcal {B}([-U,U]^3\times [0,2\pi )^3), $$

and $\mathbb {P}_0$ is the uniform probability measure in $\Omega _0$.

The $(\textsf{A},\alpha )$, $(\textsf{B},\beta )$ and $(\textsf{C},\gamma )-$flows are defined respectively by

$$\begin{aligned} f_{(\textsf{A},\alpha )}(x,y,z) = \begin{pmatrix} x + \textsf{A}\sin (z+\alpha ) \\ y + \textsf{A}\cos (z+\alpha ) \\ z \end{pmatrix} ,\\ f_{(\textsf{B},\beta )}(x,y,z) =\begin{pmatrix} x \\ y + \textsf{B}\sin (x+\beta ) \\ z + \textsf{B}\cos (x+\beta ) \end{pmatrix},\\ f_{(\textsf{C},\gamma )}(x,y,z) = \begin{pmatrix} x + \textsf{C}\cos (y+\gamma ) \\ y \\ z + \textsf{C}\sin (y+\gamma ) \end{pmatrix}. \end{aligned}$$

At the *i*-th iteration (without loss of generality we take it to be of duration 1), we make a random choice of parameters $\omega _i=(\textsf{A}_i,\textsf{B}_i,\textsf{C}_i,\alpha _i,\beta _i,\gamma _i)\in \Omega _0$ and apply the maps $f_{(\cdot ,\cdot )}$ to obtain the composition

$$ f_{\omega _i}(x,y,z) = \left( f_{(\textsf{C}_i,\gamma _i)}\circ f_{(\textsf{B}_i,\beta _i)}\circ f_{(\textsf{A}_i,\alpha _i)}\right) (x,y,z). $$

The corresponding velocity vector *u*, which depends on the noise path $\underline{\omega }=(\omega _1,\omega _2,\ldots )\in \Omega = \Omega _0^\mathbb {N}$, is given by

1.8 $$\begin{aligned} \begin{aligned} u (t,\boldsymbol{x},\underline{\omega })&= \displaystyle \sum _{i=0}^\infty \begin{pmatrix} \textsf{A}_i \sin (z+\alpha _i) \\ \textsf{A}_i \cos (z+\alpha _i) \\ 0 \end{pmatrix} \chi _{\left[ 3i,3i+1\right) }(t) + \sum _{i=0}^\infty \begin{pmatrix} 0 \\ \textsf{B}_i \sin (x+\beta _i) \\ \textsf{B}_i \cos (x+\beta _i) \end{pmatrix} \chi _{\left[ 3i+1,3i+2\right) }(t) \\&\displaystyle \quad + \sum _{i=0}^\infty \begin{pmatrix} \textsf{C}_i \cos (y+\gamma _i) \\ 0 \\ \textsf{C}_i \sin (y+\gamma _i) \end{pmatrix} \chi _{\left[ 3i+2,3i+3\right) }(t), \end{aligned} \end{aligned}$$

with $\chi _{[a,b)}(t)$ denoting the characteristic function of an interval [*a*, *b*) in the real line. Observe that even though this vector field is not smooth in *t*, it can be turned into a smooth vector field with the addition of a suitable mollifying “switching” function.

In the time discrete setting presented here, the (inviscid) flow $\phi _t$ is recovered for $t=3n\in \mathbb {N}$ by

1.9 $$\begin{aligned} \phi _{3n}(\boldsymbol{x}) = f_{\underline{\omega }}^n(\boldsymbol{x}) = f_{\omega _n}\circ f_{\omega _{n-1}}\circ \ldots \circ f_{\omega _1}(\boldsymbol{x}). \end{aligned}$$

Compared to the classical stationary ABC flows, the randomized version exhibits markedly different dynamical properties. In particular, for certain choices of parameters $\textsf{A},\textsf{B},\textsf{C}\in \mathbb {R}$, the classical ABC flows are integrable and hence non-chaotic. By contrast, the randomized version is specifically designed to enforce strong chaotic behavior, thereby ruling out the existence of integrable regimes.

The main results of this article are that *u* is an exponentially mixing vector field on $\mathbb {T}^3$, uniformly in the diffusivity parameter $\kappa \ge 0$, and is also an example of an ideal dynamo. We begin by stating the result on passive scalars.

#### Theorem 1

Let $(\phi _t)_{t\ge 0}$ be the flow defined by the SDE (1.6) with $\kappa \ge 0$ and *u* the random ABC vector field (1.8). For any q,s>0 there exist a random constant $\widehat{D}_{\underline{\omega },\kappa }\ge 0$ and a deterministic, κ-independent constant $\lambda _s>0$ such that for all mean free $g,h\in H^s(\mathbb {T}^3)$, there holds

$$ \left| \int _{\mathbb {T}^3} g(\boldsymbol{x})h(\phi _t(\boldsymbol{x}))\textrm{d}\boldsymbol{x}\right| \le \widehat{D}_{\underline{\omega },\kappa }\Vert g\Vert _{\dot{H}^s} \Vert h\Vert _{\dot{H}^s}\textrm{e}^{-\lambda _s t}, $$

almost surely for all t>0. Moreover, there exists a κ-independent constant $\overline{D}_q>0$ such that

$$ \mathbb {E}_\mathbb {P}|\widehat{D}_{\cdot ,\kappa }|^q\le \overline{D}_q. $$

The proof of this theorem is presented in Section 3, and it is based on ideas first introduced in [11] for the purely transport case κ=0, and recently extended to the advection-diffusion equation κ>0 in [19].

The result in Theorem 1 gives a decay of the so-called *correlations* between any $H^s$ and mean free functions *g* and *h*. This is a standard definition of mixing in ergodic theory, and it yields the following consequences for the passive scalar ρ that solves (1.2).

#### Corollary 1.1

Let ρ be a solution to the advection-diffusion equation (1.2) transported by the random ABC vector field (1.8) with initial configuration $\rho _0\in H^s(\mathbb {T}^3)$ and mean free. Using the notation from Theorem 1, we obtain the following estimates.

- Exponential mixing $(\kappa \ge 0)$: For any s>0,
  1.10 $$\begin{aligned} \Vert \rho (t)\Vert _{\dot{H}^{-s}} \le \widehat{D}_{\underline{\omega },\kappa }\Vert \rho _0\Vert _{\dot{H}^s}\textrm{e}^{-\lambda _s t}, \end{aligned}$$
  for every t≥0.
- Enhanced dissipation (κ>0): For any s>0,
  1.11 $$\begin{aligned} \Vert \rho (t)\Vert _{L^2} \le \frac{\widehat{D}_{\underline{\omega },\kappa }}{\kappa ^{\frac{3}{2}+s}}\Vert \rho _0\Vert _{L^2}\textrm{e}^{-\lambda _s t}, \end{aligned}$$
  for every t≥0.

The decay of correlations from Theorem 1 implies the decay of all $\dot{H}^{-s}$ norms (mixing)in (1.10) via duality. In particular, Corollary 1.1 shows that (1.8) is an example of a smooth almost-sure exponential mixer in $\mathbb {T}^3$, which is the fastest possible mixing rate for Lipschitz vector fields.

Corollary 1.1 also showcases that the vector field (1.8) enhances dissipation, see (1.11). If κ>0, mean free solutions to advection-diffusion equations with divergence free vector fields display an exponential decay of their $L^2$ norm. In general, the effect of diffusion makes the $L^2$ norm of the passive scalar to be halved in a timescale $O(\kappa ^{-1})$. On the other hand, the presence of a mixing velocity *u* enhances dissipation [17, 20], and the example of Corollary 1.1 shows that the halving time of $\Vert \rho (t)\Vert _{L^2}$ is $O(|\log \kappa |)$ – which is *optimal* at this regularity level [40]. For a proof of the enhanced dissipation estimate given uniform–in–κ decay of correlations, and other examples see [8, 19, 41].

For passive vectors, we dwell with the non-resistive setting κ=0.

#### Theorem 2

Let κ=0, $p\in [1,\infty ]$, and consider an initial datum $0\ne B_0\in L^p(\mathbb {T}^3)$. Then there exist a deterministic constant η>0, and a $\mathbb {P}-$almost surely positive random constant $\hat{k}_{\underline{\omega }}>0$ such that the solution $B(t,\boldsymbol{x})$ to (1.4) satisfies

$$ \Vert B(t)\Vert _{L^p} \ge \hat{k}_{\underline{\omega }}\textrm{e}^{\eta t}, $$

for all t≥0. In particular, the random ABC vector field *u* (1.8) is an ideal dynamo with probability 1.

The idea of considering stochastic vector fields arises naturally in the study of quantities such as the Lyapunov exponents of the flow. This approach has been explored in various contexts, including Gaussian random vector fields [7], the Kraichnan-Kazantsev model [44] – which is based on Kraichnan’s turbulence model for passive scalars–and the ABC flow with fluctuating coefficients [43].

While the positivity of the top Lyapunov exponent is a necessary condition for the fast dynamo mechanism [45], it is by no means sufficient. Nevertheless, in the ideal dynamo setting, a positive top Lyapunov exponent allows for the construction of specific initial configurations that exhibit exponential growth. A key contribution of this work is the construction of a vector field that, with probability one, induces exponential growth for *every non-trivial initial datum* in $L^p$. The result stated in Theorem 2, derived using the techniques developed for Theorem 1, represents, to the best of our knowledge, the first rigorous proof that ABC flows act as an ideal kinematic dynamo. The case with magnetic diffusivity remains an open problem.

### Organization of the paper

In Section 2 we introduce some concepts and preliminary results needed to prove Theorems 1 and 2. In particular we define three Markov chains in terms of the flow generated by the vector field (1.8), we present a suitable version of Harris Theorem about the ergodicity of the Markov chains, and we introduce some notions from the theory of random dynamics that will be key to prove our results. In Section 3 we prove the dynamical properties required for the random ABC vector field to be an exponential mixer. This includes showing topological irreducibility, aperiodicty and small sets of the different Markov chains, as well as the positivity of the Lyapunov exponent of the flow defined by *u*. Finally, in Section 4, as a byproduct of the ergodic properties of the random flow, we prove Theorem 2.

## Harris theorem and preliminary results

In this section we introduce preliminary notions and results that will be important to prove Theorems 1 and 2. First of all we introduce three different Markov transition kernels that describe the dynamics of the inviscid flow $f_\omega$ generated by the stochastic ABC vector field (1.8).

1. *The one-point chain* in $\mathbb {T}^3$. It describes the probability of a particle that started at position $\boldsymbol{x}\in \mathbb {T}^3$, to be in a Borel set $A\in \mathcal {B}(\mathbb {T}^3)$ after one full iteration of the flow map,
  $$ P(\boldsymbol{x},A) = \mathbb {P}_0[f_\omega (\boldsymbol{x})\in A]. $$
2. *The projective chain* in $\mathbb {T}^3\times \mathbb {S}^2$. It follows the position $\boldsymbol{x}\in \mathbb {T}^3$ and direction of movement $\boldsymbol{v}\in \mathbb {S}^2$ of a single particle. For this consider a Borel set $\hat{A}\in \mathcal {B}(\mathbb {T}^3\times \mathbb {S}^2)$, then we define the projective Markov chain as
  $$ \hat{P}((\boldsymbol{x},\boldsymbol{v}),\hat{A})= \mathbb {P}_0\left[ \left( f_\omega (\boldsymbol{x}),\frac{D_{\boldsymbol{x}} f_\omega \boldsymbol{v}}{|D_{\boldsymbol{x}} f_\omega \boldsymbol{v}|}\right) \in \hat{A} \right] . $$
3. *The two-point chain* in $(\mathbb {T}^3\times \mathbb {T}^3)\setminus \Delta$. Here $\Delta = \{(\boldsymbol{x}^1,\boldsymbol{x}^2)\in \mathbb {T}^3\times \mathbb {T}^3\mid \boldsymbol{x}^1=\boldsymbol{x}^2\}$ represents the diagonal in the product space $\mathbb {T}^3\times \mathbb {T}^3$. The two-point chain gives information about the position of two particles starting at $\boldsymbol{x}^1\in \mathbb {T}^3$ and $\boldsymbol{x}^2\in \mathbb {T}^3$ respectively, with $\boldsymbol{x}^1\ne \boldsymbol{x}^2$. With this notation in hand, given any Borel set $A^{(2)}\in \mathcal {B}((\mathbb {T}^3\times \mathbb {T}^3)\setminus \Delta )$, and any $(\boldsymbol{x}^1,\boldsymbol{x}^2)\in (\mathbb {T}^3\times \mathbb {T}^3)\setminus \Delta$, we define the two-point Markov chain via
  $$ P^{(2)}((\boldsymbol{x}^1,\boldsymbol{x}^2),A^{(2)})= \mathbb {P}_0\left[ \left( f_\omega (\boldsymbol{x}^1),f_\omega (\boldsymbol{x}^2)\right) \in A^{(2)} \right] . $$

In the following section we will introduce some basic theory about the Markov trasition kernel that will be applicable for the three different chains: one-point, projective and two-point. We present the results in a more abstract setting so that they are applicable for the three of them. To do so, we denote the ambient space by $\mathcal {X}$, and we assume it to be a complete metric space.

### Markov chains and Harris theorem

Let *P* be a Markov transition kernel on $\mathcal {X}$. In what comes next we will denote by $\mathcal {B}(\mathcal {X})$ the set of Borel measurable sets on $\mathcal {X}$, and $\mathcal {P}(\mathcal {X})$ the set of Borel probability measures on $\mathcal {X}$. In addition $\chi _A$ denotes the characteristic function of $A\in \mathcal {B}(\mathcal {X})$. With this notation in mind, we introduce the following features of Markov transition kernels.

- $P(\boldsymbol{x},\cdot )$ is a probability measure on $\mathcal {X}$ for each $\boldsymbol{x}\in \mathcal {X}$.
- Iterations of the Markov transition kernel are defined via Chapman-Kolmogorov,
  $$ P^{n+1}(\boldsymbol{x},A) = \int _{\mathcal {X}} P^n(\boldsymbol{z},A)P(\boldsymbol{x},\textrm{d}\boldsymbol{z}), $$
  for all $n\in \mathbb {N}$ and Borel $A\in \mathcal {B}(\mathcal {X})$.
- *P* acts on bounded Borel measurable functions $h:\mathcal {X}\rightarrow \mathbb {R}$ via
  $$ Ph(\boldsymbol{x}) = \int _{\mathcal {X}}h(\boldsymbol{z})P(\boldsymbol{x},\textrm{d}\boldsymbol{z}). $$
  If $Ph(\boldsymbol{x})$ is a continuous and bounded function on $\mathcal {X}$ for any $h(\boldsymbol{x})$ continuous and bounded, we say that *P* has the *Feller property*.
- *P* acts on probability measures μ on $\mathcal {X}$ via
  $$ P\mu (A) = \int _{\mathcal {X}} P(\boldsymbol{x},A)\textrm{d}\mu (\boldsymbol{x}), $$
  for any Borel $A\in \mathcal {B}(\mathcal {X})$.

#### Definition 2.1

We say that a probability measure $\mu \in \mathcal {P}(\mathcal {X})$ is *stationary* for the Markov transition kernel *P* if it is a fixed point for its action on the space of probability measures, namely $\mu (A)=P\mu (A)$ for any $A\in \mathcal {B}(\mathcal {X})$.

#### Definition 2.2

We say that a probability measure $\mu \in \mathcal {P}(\mathcal {X})$ is *ergodic stationary* for the Markov transition kernel *P* if all Borel sets $A\in \mathcal {B}(\mathcal {X})$ for which $P\chi _A(\boldsymbol{x})=\chi _A(\boldsymbol{x})$ μ-a.e. satisfy μ(A)=0 or μ(A)=1.

It is a classical result that if a Markov transition kernel *P* has a unique stationary measure μ, then μ is ergodic stationary, see for instance [23, Theorem 3.2.6].

Next, we introduce a version of Harris theorem that is convenient of our purposes in this paper. In order to do so, let us define the following space of weighted bounded functions. Given any function $V:\mathcal {X}\rightarrow [1,\infty )$, we say that $\phi \in L^\infty _V(\mathcal {X})$ if ϕ is measurable and

$$ \Vert \phi \Vert _{L^\infty _V} = \sup _{\boldsymbol{x}\in \mathcal {X}}\frac{|\phi (\boldsymbol{x})|}{V(\boldsymbol{x})}<\infty . $$

For a proof of this particular version of this theorem we refer to [11, Appendix A]. For a proof of more general and other versions we refer to [28, 37].

#### Theorem 3

(Harris theorem) Let *P* be a Markov transition kernel on $\mathcal {X}$ with the Feller property that possesses the following attributes.

1. (Topological irreducibility) For all $\boldsymbol{x}\in \mathcal {X}$ and all $A\subset \mathcal {X}$ open and nonempty, there exists $n\in \mathbb {N}$ such that $P^n(\boldsymbol{x},A)>0$.
2. (Small set) There exists $n\in \mathbb {N}$, an open set $A\subset \mathcal {X}$, and a positive measure $\nu _n$ in $\mathcal {X}$ such that for all $\boldsymbol{x}\in A$ there holds
  $$ P^n(\boldsymbol{x},B) \ge \nu _n(B) $$
  for all $$B\in \mathcal {B}(\mathcal {X})$$.
3. (Aperiodicity) There exists $\boldsymbol{x}^\star \in \mathcal {X}$ such that for all $A\in \mathcal {X}$ open and containing $\boldsymbol{x}^\star$ there holds that $P(\boldsymbol{x}^\star ,A)>0$.
4. (Lyapunov drift condition) There exists a measurable function $V:\mathcal {X}\rightarrow [1,\infty )$ with the following property: there exist $\alpha \in (0,1)$, β>0, and a compact set $K\subset \mathcal {X}$ such that
  $$ PV(\boldsymbol{x}) \le \alpha V(\boldsymbol{x})+\beta \chi _K(\boldsymbol{x}) $$
  for all $$\boldsymbol{x}\in \mathcal {X}$$.

Then *P* is V-uniformly geometrically ergodic, namely there exists a unique stationary measure μ for *P*, and there exist C>0 and $\gamma \in (0,1)$ such that

$$ \left| P^n\phi (\boldsymbol{x})-\int _{\mathcal {X}}\phi (\boldsymbol{x})\textrm{d}\mu (\boldsymbol{x}) \right| \le CV(\boldsymbol{x})\Vert \phi \Vert _{L^\infty _V}\gamma ^n $$

for all $\boldsymbol{x}\in \mathcal {X}$, all $\phi \in L^\infty _V(\mathcal {X})$ and all $n\in \mathbb {N}$.

If $\mathcal {X}$ is compact, then the Lyapunov drift condition (4) is redundant, since it will always be satisfied for the trivial function $V(\boldsymbol{x})=1$. This is due to the fact that $\Vert PV\Vert _{L^\infty }<\infty$ for any $V\in L^\infty$. Choosing $K=\mathcal {X}$ (compact), $\beta = \Vert PV\Vert _{L^\infty }=1$ and any $\alpha \in (0,1)$ suffices to obtain the result. In the case of $\mathcal {X}$ compact, we will say that a Markov–Feller transition kernel *P* is *uniformly geometrically ergodic* provided that it satisfies topological irreducibility, small sets and aperiodicity conditions from Harris theorem.

### Random dynamics and Lyapunov exponents

The Markov processes introduced in the previous section are derived from continuous *random dynamical systems* (RDS for short) with independent increments. For a reference about the theory of random dynamical systems see the monograph by Arnold [3].

Consider $(\Omega _0,{\mathcal {F}}_0,\mathbb {P}_0)$ a fixed probability space, and denote its elements by $\omega \in \Omega _0$. Following the notation of Section 1.3 we introduce the composition

$$ f_{\underline{\omega }}^n(\boldsymbol{x}) = f_{\omega _n}\circ \ldots \circ f_{\omega _1}(\boldsymbol{x}), $$

where $\underline{\omega } = (\omega _1,\omega _2,\ldots )$ are elements of the product space $\Omega = \Omega _0^\mathbb {N}$. In a similar fashion, we define the product probability space $(\Omega ,{\mathcal {F}},\mathbb {P}) = (\Omega _0,{\mathcal {F}}_0,\mathbb {P}_0)^\mathbb {N}$. In addition, let $\theta :\Omega \rightarrow \Omega$ be the leftwards shift, defined by $\theta \underline{\omega }=(\omega _2,\omega _3,\ldots )$. Recall that the leftward shift is a measure preserving transformation on $(\Omega ,{\mathcal {F}},\mathbb {P})$, i.e. $\theta _\#\mathbb {P}= \mathbb {P}$, and therefore $(\Omega ,{\mathcal {F}},\mathbb {P},\theta )$ defines a measure-preserving dynamical system. We introduce the following definition.

#### Definition 2.3

We say that $f_{\underline{\omega }}^n$ is a *continuous RDS* over $(\Omega ,{\mathcal {F}},\mathbb {P},\theta )$ if there holds:

1. the mapping $f_\omega :\mathcal {X}\rightarrow \mathcal {X}$ is continuous for all $\omega \in \Omega _0$;
2. the set $\{\omega \in \Omega _0 \mid f_\omega (\boldsymbol{x})\in A\}$ is ${\mathcal {F}}_0-$measurable for all $\boldsymbol{x}\in \mathcal {X}$ and all $A\in \mathcal {B}(\mathcal {X})$;
3. $f^0_{\underline{\omega }}$ is the identity map in $\mathcal {X}$ for all $\underline{\omega }\in \Omega$;
4. $f_{\underline{\omega }}^n$ satisfies the *cocycle property*, namely
  $$ f_{\underline{\omega }}^{n+m}(\boldsymbol{x}) = f_{\theta ^m\underline{\omega }}^n\circ f_{\underline{\omega }}^m(\boldsymbol{x}) $$
  for all $n,m\in \mathbb {N}$ and all $\underline{\omega }\in \Omega$.

Markov processes defined via continuous RDS $P(\boldsymbol{x},A) = \mathbb {P}_0[f_\omega (\boldsymbol{x})\in A]$ automatically have the Feller property due to the continuity of $f_\omega :\mathcal {X}\rightarrow \mathcal {X}$.

One key ingredient to study the dynamics and long-time behavior of a system defined by a RDS $f_\omega$ is the matrix $D_{\boldsymbol{x}}f_{\underline{\omega }}^n$, that gives information about the change of position of a particle at each time step. This object can be recursively defined from the measurable mapping $D_{\boldsymbol{x}}f_\omega :\Omega _0\times \mathcal {X}\rightarrow GL_d(\mathbb {R})$, where $d=\dim \mathcal {X}$, via the composition

$$ D_{\boldsymbol{x}}f_{\underline{\omega }}^n = D_{f_{\underline{\omega }}^{n-1}(\boldsymbol{x})}f_{\omega _n} \circ \ldots \circ D_{\boldsymbol{x}}f_{\omega _1}. $$

Elements with this property are called *linear cocycles*. The long-time behavior of linear cocycles is described by the *Multiplicative Ergodic Theorem* (MET), see [3, Part II], for linear cocycles that satisfy the integrability condition

2.1 $$\begin{aligned} \int _{\Omega _0\times \mathcal {X}} (\log ^+|D_{\boldsymbol{x}}f_\omega | + \log ^+|(D_{\boldsymbol{x}}f_\omega )^{-1}|) \textrm{d}\mathbb {P}_0(\omega )\textrm{d}\mu (\boldsymbol{x}) < \infty , \end{aligned}$$

where $\log ^+ a = \max \{\log a,0\}$, and μ is a stationary measure of the Markov chain generated by the continuous RDS $f_\omega$. The MET states that there exists $r\in \{1,\ldots ,d\}$, a sequence $\lambda _1(\omega ,\boldsymbol{x})>\ldots >\lambda _r(\omega ,\boldsymbol{x})$, and a filtration of subspaces

$$ \mathbb {R}^d = F_1(\omega ,\boldsymbol{x}) \supsetneq \ldots \supsetneq F_r(\omega ,\boldsymbol{x}) \supsetneq F_{r+1}(\omega ,\boldsymbol{x})=\{0\}, $$

with the property

$$ \lambda _i(\omega ,\boldsymbol{x}) = \lim _{n\rightarrow \infty } \frac{1}{n}\log |D_{\boldsymbol{x}}f_{\underline{\omega }}^n \boldsymbol{v}|, $$

for all $\boldsymbol{v}\in F_i(\omega ,\boldsymbol{x})\setminus F_{i+1}(\omega ,\boldsymbol{x})$. The subspaces $F_i(\omega ,\boldsymbol{x})$ are usualle referred to as *Oseledets spaces*, the numbers $\lambda _i(\omega ,\boldsymbol{x})$ are called *Lyapunov exponents*, and their multiplicities are defined by

$$ m_i = \dim F_i(\omega ,\boldsymbol{x})-\dim F_{i+1}(\omega ,\boldsymbol{x}). $$

In addition, the MET ensures that if the stationary measure μ of $f_\omega$ is ergodic, then the Lyapunov exponents $\lambda _i(\omega ,\boldsymbol{x}) \equiv \lambda _i$ are constant for $\mathbb {P}_0-$a.a. $\omega \in \Omega _0$, and μ-a.e. $\boldsymbol{x}\in \mathcal {X}$.

The Lyapunov exponents are key objetcs that play a crucial role in the proofs of Theorems 1 and 2. An important property of these exponents is that its total sum is given by

$$ \lambda _\Sigma = m_1\lambda _1 + \ldots +m_r\lambda _r = \lim _{n\rightarrow \infty } \frac{1}{n}\log |\det (D_{\boldsymbol{x}}f_{\underline{\omega }}^n) |. $$

This is of particular interest for RDS defined by incompressible flows because they are volume preserving and thus $|\det (D_{\boldsymbol{x}}f_{\underline{\omega }}^n)|=1$ for all $n\in \mathbb {N}$. With this in mind we see that the top Lyapunov exponent satisfies $\lambda _1\ge 0$, and $\lambda _1>0$ if r≥2.

A strictly positive Lyapunov exponent is sometimes defined as *Lagrangian chaos*, see [9]. It is a feature that goes in favour of chaos and mixing for the passive scalar, and growth of the $L^2$ norm for the passive vector. To rule out the case in which the top Lyapunov exponent is zero we use the following criterion originally introduced by Furstenberg [26].

#### Proposition 2.4

(Furstenberg’s criterion) Assume that $f_\omega$ is a volume-preserving continuous RDS with ergodic stationary measure μ and that it satisfies (2.1). If $\lambda _1=0$, then there exists a (measurable) family of measures $\{\nu _{\boldsymbol{x}}\mid \boldsymbol{x}\in {{\,\textrm{supp}\,}}(\mu )\}$ on $\mathbb {S}^{d-1}$ such that

$$ (D_{\boldsymbol{x}}f_{\underline{\omega }}^n)_\# \nu _{\boldsymbol{x}} = \nu _{f_{\underline{\omega }}^n(\boldsymbol{x})} $$

for $\mathbb {P}-$a.a. $\underline{\omega }\in \Omega$, μ-a.e. $\boldsymbol{x}\in \mathcal {X}$ and all $n\in \mathbb {N}$.

The version of Furstenberg’s criterion here presented can be found in [9, 11]. In the following section we will introduce sufficient conditions to rule out the case $\lambda _1=0$, which together with a suitable application of Harris theorem will yield the results in Theorems 1 and 2.

### Preliminary results

In [11, 19] the authors present a collection of sufficient conditions to check the assumptions in Harris Theorem 3. For the next results we will look at $f_\omega$ as a continuous random dynamical system on $\Omega _0\times \mathcal {X}\times \mathbb {N}$, and we will assume that it satisfies the following basic hypotheses.

1. The IID noise parameters ω live in a probability space $\Omega _0=\mathbb {R}^6$ with probability measure $\textrm{d}\mathbb {P}_0(\omega ) = \rho _0(\omega )\textrm{d}\omega$, where $\textrm{d}\omega$ represents the Lebesgue measure on $\mathbb {R}^6$, and $\rho _0(\omega )$ is a density. Additionally, the mapping $(\omega ,\boldsymbol{x})\mapsto f_\omega (\boldsymbol{x})$ is $C^2(\Omega _0\times \mathcal {X})$.
2. There exist a constant $C_0>0$ such that $\mathbb {P}_0-$a.s.
  $$ |(D_{\boldsymbol{x}} f_\omega )^{-1}|\le C_0, \quad |D_{\boldsymbol{x}} f_\omega |\le C_0, \quad \text {and}\quad \Vert f_\omega \Vert _{C^2}\le C_0. $$
3. $f_\omega$ preserves the Lebesgue measure on $\mathcal {X}$ $\mathbb {P}_0-$a.s.

The next result asserts that if the two-point chain generated by the inviscid flow is geometrically ergodic, then *u* mixes almost surely any $H^s$ initial datum of the advection-diffusion equation (1.2) exponentially fast, provided that the diffusivity $\kappa \ge 0$ is sufficiently small. This result for the case κ=0 was originally proved in [11, Proposition 4.6] for a time continuous flow, and in [10, Section 7] for a discrete framework more similar to the one presented here. Recently this mixing result has been extended to the more general case including diffusion in [19, Lemma 3.3].

#### Proposition 2.5

Let $f_\omega ^\kappa$ be a continuous RDS on $\mathbb {T}^3$ defined as a solution to the SDE (1.6) with $\kappa \ge 0$. Let the two-point process of $f_\omega ^0$ be V-geometrically ergodic with $V\in L^1(\mathbb {T}^3\times \mathbb {T}^3)$. Then for any q,s>0 and any $\kappa \ge 0$ sufficiently small, there exist a random constant $\widehat{C}_{\underline{\omega },\kappa }\ge 1$, and a deterministic, κ-independent constant $\lambda _s>0$ such that for all mean free $g,h\in H^s(\mathbb {T}^3)$, there holds

$$ \left| \int _{\mathbb {T}^3} g(\boldsymbol{x})h(f_{\underline{\omega }^n}^\kappa (\boldsymbol{x}))\textrm{d}\boldsymbol{x}\right| \le \widehat{C}_{\underline{\omega },\kappa } \Vert g\Vert _{H^s} \Vert h\Vert _{H^s} \textrm{e}^{-\lambda _{s} n} \quad \text {almost surely for all } n\in \mathbb {N}. $$

Moreover, there exists a κ-independent constant $\overline{C}_q>0$ such that $\mathbb {E}| \widehat{C}_{\cdot ,\kappa }|^q\le \overline{C}_q$.

Proposition 2.5 is the key piece to obtain Theorem 1. It states that we only need to prove geometric ergodicity for the two-point process of the inviscid problem κ=0 to obtain mixing uniformly in κ. Therefore from now on we will drop the superscript κ in $f^\kappa _\omega$ and we will address exclusively the inviscid case: $f_\omega$ denotes the flow given by the vector field (1.8) evaluated at discrete times.

Since the goal is to prove ergodicity of the two-point chain $P^{(2)}$, we need to make sure that assumptions in Harris theorem are satisfied. The main obstacle will be to show the Lyapunov-drift condition, which is required because $P^{(2)}$ is defined in a non-compact space $\mathcal {X}=(\mathbb {T}^3\times \mathbb {T}^3)\setminus \Delta$, however irreducibility, small sets and aperiodicity must also be checked. Topological irreducibility for the case of the random ABC vector field will be a byproduct of proving controllability. Here we present sufficient conditions for small sets and aperiodicity. The first result, about the existence of a small set, is proved in [11, Proposition 3.1].

#### Lemma 2.6

(Sufficient condition for small sets) Assume that $f_\omega$ satisfies (H1). Assume that there exist $n\in \mathbb {N}$, $\underline{\omega }^n_\star \in \Omega _0^n$, and $\boldsymbol{x}^\star \in \mathcal {X}$ that satisfy:

1. $\exists \varepsilon ,\delta >0$ so that $\rho _0(\underline{\omega }^n)\ge \delta$ for all $\underline{\omega }^n\in \Omega _0^n$ with $|\underline{\omega }^n-\underline{\omega }_\star ^n|\le \varepsilon$;
2. $\Phi _{\boldsymbol{x}^\star }(\underline{\omega }^n) = f_{\underline{\omega }^n}({\boldsymbol{x}^\star })$ is a submersion at $\underline{\omega }^n=\underline{\omega }^n_\star$.

Then there exist $A\subset \mathcal {X}$ open, and $\nu _n$ positive and absolutely continuous with respect to Lebesgue such that for all $\boldsymbol{x}\in A$ there holds

$$ P^n(\boldsymbol{x},B) \ge \nu _n(B) $$

for all $$B\in \mathcal {B}(\mathcal {X})$$.

The next lemma with a sufficient condition for aperiodicity is taken from [11, Lemma 3.2].

#### Lemma 2.7

(Sufficient condition for aperiodicity) Assume that $f_\omega$ satisfies (H1). Assume that there exists $\omega _\star \in \Omega _0$ for which $f_\omega$ has a fixed point $\boldsymbol{x}^\star \in \mathcal {X}$, i.e. $f_{\omega _\star }(\boldsymbol{x}^\star )=\boldsymbol{x}^\star$. Then for any $A\subset \mathcal {X}$ open containing $\boldsymbol{x}^\star$, there holds that $P(\boldsymbol{x}^\star ,A)>0$.

#### Remark 2.8

Observe that aperiodicity is always guaranteed for the random ABC flow (1.9) provided that we allow the coefficients $(\textsf{A},\textsf{B},\textsf{C})$ take the trivial value (0, 0, 0). This choice yields a fixed point not only for the standard one-point process, but also for the projective and two-point processes.

Irreducibility, small sets and aperiodicity are sufficient conditions for exponential ergodicity of a Markov chain that is defined in a compact space. This will be the case of the one-point *P* and periodic $\hat{P}$ chains, but not the case of the two-point chain $P^{(2)}$, that presents the problem of the diagonal. In order to deal with the two-point chain we need to address the Lyapunov-drift condition. The following result from [11, Section 4.2] gives a sufficient condition for the Lyapunov-drift condition of $P^{(2)}$ to hold true.

#### Lemma 2.9

(Lyapunov-drift condition) Assume that the one-point and the projective chains are geometrically ergodic, and further that the top Lyapunov exponent is positive. There exist p>0 sufficiently small, a function $V\in L^1(\mathbb {T}^3\times \mathbb {T}^3)$, V≥1 with the property

$$ \text {dist}_\mathcal {X}(\boldsymbol{x}^1,\boldsymbol{x}^2)^{-p} \lesssim V(\boldsymbol{x}^1,\boldsymbol{x}^2) \lesssim \text {dist}_\mathcal {X}(\boldsymbol{x}^1,\boldsymbol{x}^2)^{-p}, $$

and $\varepsilon \in (0,1)$ such that there holds $P^{(2)}V(\boldsymbol{x}^1,\boldsymbol{x}^2) < \varepsilon V(\boldsymbol{x}^1,\boldsymbol{x}^2)$ for all $(\boldsymbol{x}^1,\boldsymbol{x}^2)$ in the complementary of a compact set $K\subsetneq (\mathbb {T}^3\times \mathbb {T}^3)\setminus \Delta$.

All in all there is only one missing ingredient to apply this lemma: the positivity of the Lyapunov exponent, that we discuss in the next section.

To prove that the top Lyapunov exponent is positive we need first to make sure that the assumptions for the MET from Section 3.2 are satisfied. If the one-point chain satisfies Harris theorem, in particular has an ergodic stationary measure μ. Moreover, integrability condition (2.1) is automatically satisfied by imposing hypothesis (H2).

The following results arises as a sufficieint condition for the top Lyapunov exponent to be positive, and it is derived from Furstenberg’s criterion in Proposition 2.4. For simplicity, for this result we will work in the frame of the random ABC vector field specifically, namely $\mathcal {X}=\mathbb {T}^3$.

As for Lemma 2.6, given $n\in \mathbb {N}$ and $\boldsymbol{x}\in \mathbb {T}^3$, we define $\Phi _{\boldsymbol{x}}:\Omega _0^n\rightarrow \mathbb {T}^3$ by $\Phi _{\boldsymbol{x}}(\underline{\omega }^n) = f_{\underline{\omega }^n}(\boldsymbol{x})$. Additionally we define another mapping $\Psi _{\boldsymbol{x}}:\Omega _0^n\rightarrow SL_3(\mathbb {R})$ as $\Psi _{\boldsymbol{x}}(\underline{\omega }^n) = D_{\boldsymbol{x}}\Phi _{\boldsymbol{x}}$. Notice that $\Psi _{\boldsymbol{x}}$ is $C^1$, and moreover since the flow $f_\omega$ is volume-preserving, then $|\det (D_{\boldsymbol{x}}\Phi _{\boldsymbol{x}})|=|\det (D_{\boldsymbol{x}}f_{\underline{\omega }^n})|=1$ for all *n*, ω and $\boldsymbol{x}$, and hence $\Psi _{\boldsymbol{x}}(\underline{\omega }^n) \in SL_3(\mathbb {R})$.

#### Proposition 2.10

Under assumptions (H1)–(H2), let $f_\omega$ be a continuous RDS that is geometrically ergodic with stationary ergodic measure μ in $\mathbb {T}^3$. In addition, assume that there exist $n\in \mathbb {N}$, $\underline{\omega }^n_\star \in \Omega _0^n$, and $\boldsymbol{x}^\star \in \mathcal {X}$ that satisfy:

1. $\exists \varepsilon ,\delta >0$ so that $\rho _0(\underline{\omega }^n)\ge \delta$ for all $\underline{\omega }^n\in \Omega _0^n$ with $|\underline{\omega }^n-\underline{\omega }_\star ^n|\le \varepsilon$;
2. $\Phi _{\boldsymbol{x}^\star }(\underline{\omega }^n) = f_{\underline{\omega }^n}(\boldsymbol{x}^\star )$ is a submersion at $\underline{\omega }^n=\underline{\omega }^n_\star$;
3. the restriction of the linear mapping $D_{\underline{\omega }^n_\star }\Psi _{\boldsymbol{x}^\star }$ to $\ker (D_{\underline{\omega }^n_\star }\Phi _{\boldsymbol{x}^\star })$
  $$ D_{\underline{\omega }^n_\star }\Psi _{\boldsymbol{x}^\star }: \ker (D_{\underline{\omega }^n_\star }\Phi _{\boldsymbol{x}^\star }) \rightarrow T_{\Psi _{\boldsymbol{x}^\star }(\underline{\omega }^n)}SL_3(\mathbb {R}) $$
  is surjective.

Then $$\lambda _1>0$$.

The idea behind this result is to impose sufficient restrictions so that the *bad case* for which $\lambda _1=0$ in Proposition 2.4 is ruled out. For a discussion about this result see [11, Section 3.2].

At this stage we have all the tools needed to prove Theorem 1. The to-do list reads a follows.

- Check that the basic assumptions (H1)–(H3) are satisfied.
- Check topological irreducibility for the one-point, the projective and the two-point chains. Using Lemma 2.6, check that the three Markov chains satisfy the small sets condition. Aperiodicity is obtained directly due to Remark 2.8 and Lemma 2.7. Thanks to Harris Theorem 3, this yields geometric ergodicity for the one-point and projective processes since both are defined in compact spaces, $\mathbb {T}^3$ and $\mathbb {T}^3\times \mathbb {S}^2$ respectively.
- Check the positivity of the Lyapunov exponent using Proposition 2.10.
- Via Lemma 2.9, geometric ergodicity of the one-point and projective chains together with a positive Lyapunov exponent yield the Lyapunov-drift condition for the two-point chain, concluding via Harris theorem that the two-point chain is V-geometrically ergodic.

Additionally Theorem 2 will be proved using a nonrandom version of the MET, the positivity of the Lyapunov exponent and the ergodicity of the three Markov chains *P*, $\hat{P}$ and $P^{(2)}$ in Section 4.

## Almost-sure exponential mixing: proof of Theorem 1

In this section we show that the flow defined by the random ABC vector field (1.8) satisfies all the requirements to obtain almost sure exponential mixing. As noted in Remark 2.8, aperiodicity is given by allowing $\textsf{A}$, $\textsf{B}$ and $\textsf{C}$ to take the trivial value zero, so we now put a focus on proving topological irreducibility and small sets of the three Markov chains *P*, $\hat{P}$ and $P^{(2)}$.

### Topological irreducibility and small sets

We start by proving irreducibility and small sets. For the first property we will show that the three Markov chains are controllable, by making an explicit choice of parameters that take any initial point to any target point. To prove small sets we use Lemma 2.6.

#### The one-point chain

Recall that for any $A\in \mathcal {B}(\mathbb {T}^3)$ and $\boldsymbol{x}=(x,y,z)\in \mathbb {T}^3$, we define the one-point Markov transition kernel via

$$ P(\boldsymbol{x},A) = \mathbb {P}_0[f_\omega (\boldsymbol{x}) \in A]. $$

##### Lemma 3.1

Given any $\boldsymbol{x},\boldsymbol{x}^\star \in \mathbb {T}^3$, there exist $N\in \mathbb {N}$ and $\underline{\omega }^N=(\omega _1,\ldots ,\omega _N)\in \Omega _0^N$ for which there holds $f_{\underline{\omega }^N}(\boldsymbol{x})= \boldsymbol{x}^\star$, namely the one-point process is exactly controllable.

##### Proof

Exact controllability of the one-point chain is indeed achievable in one time step provided that $U\ge \pi$, i.e. the coefficients $\textsf{A}$, $\textsf{B}$ and $\textsf{C}$ can take values up to the size of the domain. If this were not the case, then one might to do more steps (a finite number) to reach the target. To see this, notice that one can choose α, β and γ such that $\cos (z+\alpha )=1$, $\cos (x+\beta )=1$ and $\cos (y+\gamma )=1$, so that there yields

$$ x^\star = x + \textsf{C}, \quad y^\star = y + \textsf{A}, \quad \text {and} \quad z^\star = z + \textsf{B}. $$

Now, since $\text {dist}_\mathbb {T}(x^\star ,x),\text {dist}_\mathbb {T}(y^\star ,y),\text {dist}_\mathbb {T}(z^\star ,z)\le \pi \le U$, there is a choice of $\textsf{A},\textsf{B},\textsf{C}\in [-U,U]$ for which $\boldsymbol{x}^\star$ is reachable in one step. □

To check that the one-point chain admits a small set we use Lemma 2.6. Observe that one must only check that the mapping $\Phi _{\boldsymbol{x}}: \Omega _0^n \rightarrow \mathbb {T}^3$ defined as

3.1 $$\begin{aligned} \Phi _{\boldsymbol{x}}(\underline{\omega }^n) = f_{\underline{\omega }^n}(\boldsymbol{x}) \end{aligned}$$

is a submersion for some $n\in \mathbb {N}$, some $\boldsymbol{x}\in \mathbb {T}^3$ and some $\underline{\omega }^n\in \Omega _0^n$. The remaining assumptions needed to apply Lemma 2.6 are trivially satisfied by the definition of the noise that we are considering in this example.

For the one-point chain notice that for n=1, the matrix $D_\omega \Phi _{\boldsymbol{x}}$ is associated to a linear mapping from $\mathbb {R}^6$ to $\mathbb {R}^3$. For instance, we can select

$$ \boldsymbol{x}=(0,0,0), \quad (\textsf{A},\textsf{B},\textsf{C})=(0,0,0), \quad (\alpha ,\beta ,\gamma )=\left( \frac{\pi }{2},\frac{\pi }{2},\frac{\pi }{2}\right) , $$

where $\omega =(\textsf{A},\textsf{B},\textsf{C},\alpha ,\beta ,\gamma )$. Then we obtain the matrix

$$ D_\omega \Phi _{\boldsymbol{x}} = \begin{pmatrix} 1 &  0 &  0 &  0 &  0 &  0\\ 0 &  1 &  0 &  0 &  0 &  0\\ 0 &  0 &  1 &  0 &  0 &  0\\ \end{pmatrix} $$

that has full rank. This together with irreducibility and aperiocidity, given that $\mathbb {T}^3$ is a compact domain, yields that *P* is uniformly geometric ergodic, with the Lebesgue measure as ergodic stationary measure.

#### The projective chain

Let $\hat{A}\in \mathcal {B}(\mathbb {T}^3\times \mathbb {S}^2)$, $\boldsymbol{x}=(x,y,z)\in \mathbb {T}^3$, and $\boldsymbol{v}=(v^x,v^y,v^z)\in \mathbb {S}^2$, we define the projective Markov transition kernel as

3.2 $$\begin{aligned} \hat{P}((\boldsymbol{x},\boldsymbol{v}),\hat{A})= \mathbb {P}_0\left[ \left( f_\omega (\boldsymbol{x}),\frac{D_{\boldsymbol{x}} f_\omega \boldsymbol{v}}{|D_{\boldsymbol{x}} f_\omega \boldsymbol{v}|}\right) \in \hat{A} \right] . \end{aligned}$$

The matrix $D_{\boldsymbol{x}} f_\omega$ is a 3×3 real matrix that has the following entries,

$$\begin{aligned} {[}D_{\boldsymbol{x}} f_\omega ]_{11}&= 1 - \textsf{B}\textsf{C}\cos (\beta + x + \textsf{A}\sin (\alpha + z))  &   \\&\qquad \times \sin ( \gamma + y + \textsf{A}\cos (\alpha + z) + \textsf{B}\sin (\beta + x + \textsf{A}\sin (\alpha + z))),  &   \end{aligned}$$

$$\begin{aligned} {[}D_{\boldsymbol{x}} f_\omega ]_{12} = -\textsf{C}\sin (\gamma + y + \textsf{A}\cos (\alpha + z) + \textsf{B}\sin (\beta + x + \textsf{A}\sin (\alpha + z))),  &   \end{aligned}$$

$$\begin{aligned} {[}D_{\boldsymbol{x}} f_\omega ]_{13}&= \textsf{A}\cos (\alpha + z) - \textsf{C}(\textsf{A}\textsf{B}\cos (\alpha + z) \cos (\beta + x + \textsf{A}\sin (\alpha + z)) - \textsf{A}\sin (\alpha + z))  &   \\&\qquad \times \sin ( \gamma + y + \textsf{A}\cos (\alpha + z) + \textsf{B}\sin (\beta + x + \textsf{A}\sin (\alpha + z))),  &   \end{aligned}$$

$$\begin{aligned} {[}D_{\boldsymbol{x}} f_\omega ]_{21} = \textsf{B}\cos (\beta + x + \textsf{A}\sin (\alpha + z)),  &   \end{aligned}$$

$$\begin{aligned} {[}D_{\boldsymbol{x}} f_\omega ]_{22} = 1,  &   \end{aligned}$$

$$\begin{aligned} {[}D_{\boldsymbol{x}} f_\omega ]_{23} = \textsf{A}\textsf{B}\cos (\alpha + z) \cos (\beta + x + \textsf{A}\sin (\alpha + z)) - \textsf{A}\sin (\alpha + z),  &   \end{aligned}$$

$$\begin{aligned} {[}D_{\boldsymbol{x}} f_\omega ]_{31}&= \textsf{B}\textsf{C}\cos (\beta + x + \textsf{A}\sin (\alpha + z))  &   \\&\qquad \times \cos ( \gamma + y + \textsf{A}\cos (\alpha + z) + \textsf{B}\sin (\beta + x + \textsf{A}\sin (\alpha + z)))  &   \\&\qquad - \textsf{B}\sin (\beta + x + \textsf{A}\sin (\alpha + z)),  &   \end{aligned}$$

$$\begin{aligned} {[}D_{\boldsymbol{x}} f_\omega ]_{32} = \textsf{C}\cos (\gamma + y + \textsf{A}\cos (\alpha + z) + \textsf{B}\sin (\beta + x + \textsf{A}\sin (\alpha + z))),  &   \end{aligned}$$

$$\begin{aligned} {[}D_{\boldsymbol{x}} f_\omega ]_{33}&= 1 + \textsf{C}\cos ( \gamma + y + \textsf{A}\cos (\alpha + z) + \textsf{B}\sin (\beta + x + \textsf{A}\sin (\alpha + z)))  &   \\&\qquad \times (\textsf{A}\textsf{B}\cos (\alpha + z) \cos ( \beta + x + \textsf{A}\sin (\alpha + z)) - \textsf{A}\sin (\alpha + z))  &   \\&\qquad - \textsf{A}\textsf{B}\cos (\alpha + z) \sin (\beta + x + \textsf{A}\sin (\alpha + z)).  &   \end{aligned}$$

Even if componentwise this matrix seems certainly cluttered, there is some hidden structure. Let us introduce the following variables,

3.3 $$\begin{aligned} Z = \alpha + z, \quad X=\beta +x+\textsf{A}\sin Z, \quad Y = \gamma + y + \textsf{A}\cos Z + \textsf{B}\sin X, \end{aligned}$$

then we can write the matrix $D_{\boldsymbol{x}} f_\omega$ as

$$ D_{\boldsymbol{x}} f_\omega = \begin{pmatrix} 1-\textsf{B}\textsf{C}\cos X\sin Y &  -\textsf{C}\sin Y &  -\textsf{A}(\textsf{C}\Phi \sin Y-\cos Z)\\ \textsf{B}\cos X &  1 &  \textsf{A}\Phi \\ \textsf{B}(\textsf{C}\cos X\cos Y-\sin X) &  \textsf{C}\cos Y &  1+\textsf{A}(\textsf{C}\Phi \cos Y-\textsf{B}\cos Z\sin X) \end{pmatrix}, $$

where $\Phi =\textsf{B}\cos X\cos Z-\sin Z$. This matrix has the property that

$$ \det D_{\boldsymbol{x}} f_\omega = 1 $$

for all $\boldsymbol{x}\in \mathbb {T}^3$ and $\omega \in \Omega _0$. Invertibility of $D_{\boldsymbol{x}} f_\omega$ is a key feature that we will use to prove irreducibility of the projective chain. For this reason it is convenient to display the form that its inverse matrix has. Using the short hand notation (3.3), we find that

$$ (D_{\boldsymbol{x}} f_\omega )^{-1} = \begin{pmatrix} 1-\textsf{A}\textsf{B}\sin X\cos Z &  -\textsf{C}(\textsf{A}\Psi \cos Z-\sin Y) &  -\textsf{A}\cos Z\\ \textsf{B}(\textsf{A}\sin X\sin Z-\cos X) &  1+\textsf{C}(\textsf{A}\Psi \sin Z-\textsf{B}\cos X\sin Y) &  \textsf{A}\sin Z \\ \textsf{B}\sin X &  \textsf{C}\Psi &  1\\ \end{pmatrix}, $$

where $$\Psi = \textsf{B}\sin X\sin Y - \cos Y$$.

To show topological irreducibility of the projective Markov chain we will prove that, as the one-point chain, it is exactly controllable.

##### Proposition 3.2

Given any two pairs $(\boldsymbol{x},\boldsymbol{v}),(\boldsymbol{x}^\star ,\boldsymbol{v}^\star )\in \mathbb {T}^3\times \mathbb {S}^2$, there exist $N\in \mathbb {N}$ and $\underline{\omega }^N=(\omega _1,\ldots ,\omega _N)\in \Omega _0^N$ such that

$$ f_{\underline{\omega }^N}(\boldsymbol{x}) = \boldsymbol{x}^\star \quad \text {and} \quad \frac{D_{\boldsymbol{x}} f_{\underline{\omega }^N}\boldsymbol{v}}{|D_{\boldsymbol{x}} f_{\underline{\omega }^N}\boldsymbol{v}|}= \boldsymbol{v}^\star . $$

The proof of this proposition can be split into three different parts. First of all, we prove that the vector $\overline{\boldsymbol{v}} = (1,0,0)$ is reachable from any $\boldsymbol{v}\in \mathbb {S}^2$. Second, we prove that we can get to any given $\overline{\boldsymbol{x}}\in \mathbb {T}^3$ from any point $\boldsymbol{x}\in \mathbb {T}^3$ only via *rigid motions*, namely keeping $\overline{\boldsymbol{v}} = (1,0,0)$ fixed. Last, we show that we can go exactly from $\overline{\boldsymbol{v}} = (1,0,0)$ to the target $\boldsymbol{v}^\star$ in such a way that we end up exactly the target point $\boldsymbol{x}^\star$ for the dynamical system in $\mathbb {T}^3$.

Let us introduce the short notation defined recursively via

$$ \boldsymbol{x}_n = f_{\omega _n}(\boldsymbol{x}_{n-1})\in \mathbb {T}^3, \quad \boldsymbol{v}_n = \frac{D_{\boldsymbol{x}_{n-1}}f_{\underline{\omega }^n}\boldsymbol{v}_{n-1}}{|D_{\boldsymbol{x}_{n-1}}f_{\underline{\omega }^n}\boldsymbol{v}_{n-1}|}\in \mathbb {S}^2, $$

with $\boldsymbol{x}_0=\boldsymbol{x}$ and $\boldsymbol{v}_0=\boldsymbol{v}$. The first lemma we want to introduce displays the fact the second component of the projective process, denoted by $\boldsymbol{v}_n$, is also exactly controllable.

##### Lemma 3.3

Let $\boldsymbol{v}\in \mathbb {S}^2$. The following results hold true.

1. There exist $N_1\in \mathbb {N}$ and $\underline{\omega }^{N_1}=(\omega _1,\ldots ,\omega _{N_1})\in \Omega _0^{N_1}$ such that $\boldsymbol{v}_{N_1} = (1,0,0)$.
2. If initially $\boldsymbol{v}= (1,0,0)$, then given any $\overline{\boldsymbol{v}}\in \mathbb {S}^2$, there exist $N_2\in \mathbb {N}$ and $\underline{\omega }^{N_2}=(\omega _1,\ldots ,\omega _{N_2})\in \Omega _0^{N_2}$ such that $\boldsymbol{v}_{N_2}=\overline{\boldsymbol{v}}$.

##### Proof

The first point that we will show is that the process $\boldsymbol{v}_n$ can actually reach one of the poles in one time step. Since in the claim we are choosing specifically the pole (1, 0, 0), we will show that anyways one can reach this pole in at most two time steps from any starting point $\boldsymbol{v}\in \mathbb {S}^2$. Fix $\boldsymbol{v}\in \mathbb {S}^2$ and $\boldsymbol{x}\in \mathbb {T}^3$, then notice that we look for a choice of $\omega \in \Omega _0$ and some $k\in \mathbb {R}$, k≠0, so that

$$ \boldsymbol{v}= \begin{pmatrix} v^x \\ v^y \\ v^z\\ \end{pmatrix} = (D_{\boldsymbol{x}} f_\omega )^{-1} \begin{pmatrix} k \\ 0 \\ 0\\ \end{pmatrix} = k \begin{pmatrix} 1-\textsf{A}\textsf{B}\sin X\cos Z \\ \textsf{B}(\textsf{A}\sin X\sin Z-\cos X) \\ \textsf{B}\sin X\\ \end{pmatrix}, $$

where we use the variables introduced in (3.3). We can choose $\alpha \in [0,2\pi )$ so that $\cos (\alpha +z)=1$, and hence

$$ \begin{pmatrix} v^x \\ v^y \\ v^z\\ \end{pmatrix}= k \begin{pmatrix} 1-\textsf{A}\textsf{B}\sin (\beta +x)) \\ -\textsf{B}\cos (\beta +x) \\ \textsf{B}\sin (\beta +x)\\ \end{pmatrix}. $$

Now, if $v^z\ne 0$, choose k=1, $\textsf{B}\ne 0$ and $\beta \in [0,2\pi )$ so that $v^y={-}\textsf{B}\cos (\beta +x)$ and $v^z=\textsf{B}\sin (\beta +x)$, and choose $\textsf{A}$ such that $v^x=1-\textsf{A}v^z$. However, if $v^z=0$ and $v^x\ne 0$, choose β so that $\sin (\beta +x)=0$, and $\textsf{B}$ so that the vector $(1,-\textsf{B},0)$ is parallel to $(v^x,v^y,0)$.

It is clear that the only situation that is not achievable in one time step and using this choice of parameters is $v^x=v^z=0$. Arguably this is not a problem because this would be one of the degenerate situations that we are looking for. However, for the sake of completion we will show that one can also take the vector (0, 1, 0) to (1, 0, 0) in two time steps. To see this, notice that

$$ D_{\boldsymbol{x}} f_\omega \begin{pmatrix} 0\\ 1 \\ 0\\ \end{pmatrix} = \begin{pmatrix} -\textsf{C}\sin (\gamma + y + \textsf{A}\cos (\alpha + z) + \textsf{B}\sin (\beta + x + \textsf{A}\sin (\alpha + z))) \\ 1 \\ \textsf{C}\cos (\gamma + y + \textsf{A}\cos (\alpha + z) + \textsf{B}\sin (\beta + x + \textsf{A}\sin (\alpha + z)))\\ \end{pmatrix}, $$

hence it is clear that one can choose $\omega \in \Omega _0$ so that the first component is not vanishing. Now, repeating the previous argument for the case $v^x\ne 0$ and there yields the claim of (1).

To prove (2), we argue in a similar fashion. First of all, notice that

3.4 $$\begin{aligned} D_{\boldsymbol{x}} f_\omega \begin{pmatrix} 1 \\ 0 \\ 0 \end{pmatrix} = \begin{pmatrix} 1-\textsf{B}\textsf{C}\cos X\sin Y\\ \textsf{B}\cos X \\ \textsf{B}(\textsf{C}\cos X\cos Y-\sin X) \end{pmatrix}. \end{aligned}$$

In order to simplify the computations, let us choose as before $\alpha \in [0,2\pi )$ so that $\cos (\alpha +z)=1$,

$$ D_{\boldsymbol{x}} f_\omega \begin{pmatrix} 1 \\ 0 \\ 0 \end{pmatrix} = \begin{pmatrix} 1 - \textsf{B}\textsf{C}\cos (\beta + x) \sin (\textsf{A}+ \gamma + y + \textsf{B}\sin (\beta + x)) \\ \textsf{B}\cos (\beta +x) \\ \textsf{B}\textsf{C}\cos (\beta + x) \cos (\textsf{A}+ \gamma + y + \textsf{B}\sin (\beta + x)) - \textsf{B}\sin (\beta + x) \end{pmatrix}. $$

The goal is to reach the target vector $\boldsymbol{v}=(v^x,v^y,v^z)$, however this might not be possible in one time step since $\textsf{A},\textsf{B},\textsf{C}\in [-U,U]$. The strategy will be to take an intermediate step in the vector $\overline{\boldsymbol{v}}=(1,1,1)/\sqrt{3}$. Let us choose $\beta \in [0,2\pi )$ so that $\cos (\beta +x)=1$, hence we look for parameters $\textsf{A}$, $\textsf{B}$, γ and *k* such that

$$ \begin{pmatrix} 1 - \textsf{B}\textsf{C}\sin (\textsf{A}+ \gamma + y) \\ \textsf{B}\\ \textsf{B}\textsf{C}\cos (\textsf{A}+ \gamma + y) \end{pmatrix} = k \begin{pmatrix} 1 \\ 1 \\ 1 \end{pmatrix}. $$

This is an admissible step since, for instance, k=1, $\textsf{B}=1$, $\textsf{C}=1$ and $\textsf{A}+\gamma$ such that $\cos (\textsf{A}+\gamma +y)=1$, yields a solution.

On a second time step we want to reach the target vector $\boldsymbol{v}=(v^x,v^y,v^z)$ from $\overline{\boldsymbol{v}}=(1,1,1)/\sqrt{3}$. Now it is more convenient to consider the inverse map from $\boldsymbol{v}$. To simplify the computations as before, we choose α and β so that $\cos (\alpha +z)=1$ and $\cos (\beta +x)=1$, and in addition now we impose $\textsf{A}=0$, then we obtain

$$ (D_{\boldsymbol{x}} f_\omega )^{-1} \begin{pmatrix} v^x \\ v^y \\ v^z\\ \end{pmatrix} = \begin{pmatrix} v^x + \textsf{C}\sin (\gamma + y)v^y\\ -\textsf{B}v^x + (1 - \textsf{B}\textsf{C}\sin (\gamma + y))v^y \\ v^z - \textsf{C}\cos (\gamma + y)v^y \end{pmatrix}. $$

Let us assume for the moment that $v^y\ne 0$, and notice that for the mapping

$$ \boldsymbol{w}= \begin{pmatrix} w^x \\ w^y \\ w^z \end{pmatrix} = (D_{\boldsymbol{x}} f_\omega )^{-1} \begin{pmatrix} v^x \\ v^y \\ v^z \end{pmatrix} = \begin{pmatrix} v^x + \textsf{C}\sin (\gamma + y)v^y\\ -\textsf{B}v^x + (1 - \textsf{B}\textsf{C}\sin (\gamma + y))v^y \\ v^z - \textsf{C}\cos (\gamma + y)v^y \end{pmatrix} $$

the second component has a fixed point $w^y=v^y$ provided that $\textsf{B}=0$. Therefore, for fixed $v^y\ne 0$, there exist some $N\in \mathbb {N}$ and finite sequences $\{\textsf{C}_n\}_{1\le n\le N}\subset [-U,U]$, $\{\gamma _n\}_{1\le n\le N}\subset [0,2\pi )$, defined by

$$ \boldsymbol{w}_n = \begin{pmatrix} w^x_n \\ w^y_n \\ w^z_n \end{pmatrix} = \begin{pmatrix} w^x_{n-1} + \textsf{C}_n \sin (\gamma _n + y)v^y \\ v^y \\ w^z_{n-1} - \textsf{C}_n \cos (\gamma _n + y)v^y \end{pmatrix}, $$

with $\boldsymbol{w}_0=\boldsymbol{v}$ and such that $w^x_N=w^z_N=k$ for some $k\in \mathbb {R}$, k≠0. Notice that the coordinate *y* in these dynamics is a fixed point, it should in principle change with *n*, but it does not since $\textsf{A}_n=\textsf{B}_n=0$ for all $1\le n\le N$. Finally, one more iteration of the inverse flow map with the choice $\textsf{C}_{N+1}=0$ yields that

$$ \boldsymbol{w}_{N+1} = \begin{pmatrix} w_N^x \\ -\textsf{B}_{N+1}w_N^x + w_N^y \\ w_N^z \end{pmatrix} = \begin{pmatrix} k \\ -\textsf{B}_{N+1}k + v^y \\ k \end{pmatrix}. $$

Thus, upon choosing $\textsf{B}_{N+1} = v^yk^{-1}-1$ we arrive at

$$ \boldsymbol{w}_{N+1} = k \begin{pmatrix} 1 \\ 1 \\ 1 \end{pmatrix}, $$

which concludes the proof of the lemma for all target points $\boldsymbol{v}$ with second component $v^y\ne 0$. For the remaining case just notice that we can take a preliminary step where now we do not assume $\textsf{A}=0$ as before and define

$$ \boldsymbol{w}_0 = (D_{\boldsymbol{x}} f_\omega )^{-1} \begin{pmatrix} v^x \\ 0 \\ v^z \end{pmatrix} = \begin{pmatrix} v^x - \textsf{A}v^z \\ -\textsf{B}v^x \\ v^z \end{pmatrix}. $$

If $v^x\ne 0$, any choice of $\textsf{B}\ne 0$ produces $w^y\ne 0$. If we are in the degenerate situation that $v^x={v^y}=0$ then $v^z=1$, so selecting $\textsf{A}\ne 0$ one produces $w^x\ne 0$. Finally, repeating the scheme designed for the case $v^y\ne 0$ we find that the proof of the lemma is complete. □

##### Lemma 3.4

Assume that $\boldsymbol{v}= (1,0,0)$. Then given any $\boldsymbol{x},\overline{\boldsymbol{x}}\in \mathbb {T}^2$, there exist $N_3\in \mathbb {N}$ and $\underline{\omega }^{N_3}=(\omega _1,\ldots ,\omega _{N_3})\in \Omega _0^{N_3}$ such that

$$ f_{\underline{\omega }^{N_3}}(\boldsymbol{x})=\overline{\boldsymbol{x}}, \quad \text {and}\quad \boldsymbol{v}_{N_3}= (1,0,0). $$

##### Proof

On the one hand, notice that if one wants keep the vector $\boldsymbol{v}=(1,0,0)$ a fixed point in the projective dynamics, it suffices to fix the parameter $\textsf{B}=0$, see (3.4). On the other hand, as it is evident from the construction of the one-point chain, even with the restriction $\textsf{B}=0$ the process is exactly controllable. To picture this, notice that if $\textsf{B}=0$ then

$$ \begin{pmatrix} x_1 \\ y_1 \\ z_1 \end{pmatrix} = \begin{pmatrix} x + \textsf{C}\cos (\gamma + y + \textsf{A}\cos (\alpha + z)) + \textsf{A}\sin (\alpha + z) \\ y + \textsf{A}\cos (\alpha + z) \\ z + \textsf{C}\sin (\gamma + y + \textsf{A}\cos (\alpha + z)) \end{pmatrix}. $$

Now choose α so that $\cos (\alpha + z)=1$ and $\textsf{A}$ such that $y+\textsf{A}=\overline{y}$. Then there yields,

$$ \begin{pmatrix} x_1 \\ y_1 \\ z_1 \end{pmatrix} = \begin{pmatrix} x + \textsf{C}\cos (\gamma + \overline{y}) \\ \overline{y} \\ z + \textsf{C}\sin (\gamma + \overline{y}) \end{pmatrix} . $$

The goal now is to reach the point $(\overline{x},\overline{z})$ from (*x*, *z*) in the plane $\{y=\overline{y}\}$. It is possible that such thing is not doable in one time step since $|\textsf{C}|\le U$, but it is clear that $(\overline{x},\overline{z})$ is reachable in a finite number of steps. In particular, the maximum distance between $(\overline{x},\overline{z})$ and (*x*, *z*) is $\sqrt{2}\pi$, then if we assume U=π, this means that with these constrains any point can be reached at most in two time steps. □

##### Proof of Proposition 3.2

Let ε>0, and fix $(\boldsymbol{x},\boldsymbol{v}),(\boldsymbol{x}^\star ,\boldsymbol{v}^\star )\in \mathbb {T}^3\times \mathbb {S}^2$. Using Lemma 3.3, there exist $N_1>0$ and $\underline{\omega }^{N_1}\in \Omega _0^{N_1}$ such that

$$ \boldsymbol{v}_{N_1} (\boldsymbol{v})= \frac{D_{\boldsymbol{x}_{N_1}}f_{\underline{\omega }^{N_1}}\boldsymbol{v}}{|D_{\boldsymbol{x}_{N_1}}f_{\underline{\omega }^{N_1}}\boldsymbol{v}|} = \overline{\boldsymbol{v}} = \begin{pmatrix} 1\\ 0\\ 0 \end{pmatrix} . $$

This process brings the point $\boldsymbol{x}\in \mathbb {T}^3$ to $\boldsymbol{x}_{N_1}\in \mathbb {T}^3$. An application of the same lemma yields that there exist $N_2>0$ and $\underline{\omega }^{N_2}\in \Omega _0^{N_2}$ so that

$$ \boldsymbol{v}_{N_2}(\overline{\boldsymbol{v}}) = \frac{D_{\boldsymbol{x}_{N_2}}f_{\underline{\omega }^{N_2}}\overline{\boldsymbol{v}}}{|D_{\boldsymbol{x}_{N_2}}f_{\underline{\omega }^{N_2}}\overline{\boldsymbol{v}}|} = \boldsymbol{v}^\star , $$

hence we define

$$ \overline{\boldsymbol{x}} = f_{\underline{\omega }^{N_2}}^{-1}(\boldsymbol{x}^\star ). $$

Lemma 3.4 implies that there exist $N_3\in \mathbb {N}$ and $\underline{\omega }^{N_3}\in \Omega _0^{N_3}$ such that

$$ f_{\underline{\omega }^{N_3}}(\boldsymbol{x}_{N_1})=\overline{\boldsymbol{x}} \quad \text {and}\quad \boldsymbol{v}_{N_3}= \begin{pmatrix} 1\\ 0\\ 0 \end{pmatrix} . $$

All in all, we set the noise vector $\underline{\omega }^N = (\underline{\omega }^{N_1},\underline{\omega }^{N_3},\underline{\omega }^{N_2})\in \Omega _0^N$, where $N=N_1+N_3+N_2$, so that we obtain $\boldsymbol{v}_N=\boldsymbol{v}^\star$ and $f_{\underline{\omega }^N}(\boldsymbol{x})=\boldsymbol{x}^\star$, and thus there yields the claim of the proposition. □

##### Remark 3.5

*(On the invertibility of the flow map)* In the proof of Proposition 3.2 we have used the inverse map $f_{\underline{\omega }^{N_2}}^{-1}(\boldsymbol{x}^\star )$. Give a noise realisation $\omega \in \Omega _0$, the inverse flow map is defined by

$$ f^{-1}_\omega (\boldsymbol{x}) = \left( f_{(\textsf{A},\alpha )}^{-1}\circ f_{(\textsf{B},\beta )}^{-1}\circ f_{(\textsf{C},\gamma )}^{-1}\right) (\boldsymbol{x}), $$

where the inverse of each of the partial applications of the flow can be presented from two different perspectives,

1. either we can choose to define
  $$ f_{(\textsf{A},\alpha )}^{-1} = f_{(\textsf{A},\alpha +\pi )}, \quad f_{(\textsf{B},\beta )}^{-1} = f_{(\textsf{B},\beta +\pi )}, \quad f_{(\textsf{C},\gamma )}^{-1} = f_{(\textsf{C},\gamma +\pi )}; $$
2. or either
  $$ f_{(\textsf{A},\alpha )}^{-1} = f_{(-\textsf{A},\alpha )}, \quad f_{(\textsf{B},\beta )}^{-1} = f_{(-\textsf{B},\beta )}, \quad f_{(\textsf{C},\gamma )}^{-1} = f_{(-\textsf{C},\gamma )}. $$

Both definitions yield that $f_{\omega }^{-1}(f_{\omega }(\boldsymbol{x})) = \boldsymbol{x}$, and moreover they fall under the theoretical frame here considered with small modifications. Therefore one can apply Lemma 3.4 as well to the inverse flow map as required in the proof of the proposition.

To show that the projective process admits a small set we find $\boldsymbol{x}$ and $\underline{\omega }^n$ for which the assumption of Lemma 2.6 are satisfied.

Given $n\in \mathbb {N}$ and $(\boldsymbol{x},\boldsymbol{v})\in \mathbb {T}^3\times \mathbb {S}^2$ we define the mapping $\hat{\Phi }_{\boldsymbol{x},\boldsymbol{v}}: \Omega _0^n \rightarrow \mathbb {T}^3\times \mathbb {S}^2$ as

3.5 $$\begin{aligned} \hat{\Phi }_{\boldsymbol{x},\boldsymbol{v}}(\underline{\omega }^n) = \left( f_{\underline{\omega }^n}(\boldsymbol{x}),\frac{D_{\boldsymbol{x}} f_{\underline{\omega }^n}\boldsymbol{v}}{|D_{\boldsymbol{x}} f_{\underline{\omega }^n}\boldsymbol{v}|}\right) . \end{aligned}$$

It is sufficient to consider the case n=1, so $D_{\omega }\hat{\Phi }_{(\boldsymbol{x},\boldsymbol{v})}$ can be understood a linear map from $\mathbb {R}^6$ to $\mathbb {R}^6$. The sufficient condition for the projective process to admit a small set then boils down to find a collection of $\boldsymbol{x}$, $\boldsymbol{v}$ and $\omega =(\textsf{A},\textsf{B},\textsf{C},\alpha ,\beta ,\gamma )$ for which $\text {rank} (D_{\omega }\hat{\Phi }_{(\boldsymbol{x},\boldsymbol{v})})\ge 5$. In this regard let us define

$$ \boldsymbol{x}=(0,0,0),\quad \boldsymbol{v}=(0,1,0),\quad (\textsf{A},\textsf{B},\textsf{C})=\left( \pi ,0,1 \right) , \quad (\alpha ,\beta ,\gamma )=(0,0,0), $$

which yields

$$ D_{\omega }\hat{\Phi }_{(\boldsymbol{x},\boldsymbol{v})} = \begin{pmatrix} 0 &  0 &  -1 &  \pi &  0 &  0\\ 1 &  0 &  0 &  0 &  0 &  0\\ -1 &  1 &  0 &  0 &  0 &  -1\\ 1/\sqrt{2} &  0 &  0 &  0 &  0 &  1/\sqrt{2}\\ 0 &  0 &  -1/(2\sqrt{2}) &  0 &  0 &  0\\ 0 &  0 &  -1/(2\sqrt{2}) &  0 &  0 &  0 \end{pmatrix}. $$

This matrix has rank 5 since the first five rows are linearly independent, thus from Lemma 2.6 it follows that the projective chain admits a small set. This result together with irreducibility and aperiodicity yields in fact that $\hat{P}$ is uniformly geometrically ergodic in $\mathbb {T}^3\times \mathbb {S}^2$.

#### The two-point chain

The two-point chain is arguably the most important object in the study of mixing. It gives information about the position of two particles starting at $\boldsymbol{x}^1=(x^1,y^1,z^1)\in \mathbb {T}^3$ and $\boldsymbol{x}^2=(x^2,y^2,z^2)\in \mathbb {T}^3$ respectively, with $\boldsymbol{x}^1\ne \boldsymbol{x}^2$. After $n\in \mathbb {N}$ iterations in which both particles are advected by the same vector field with the same noise realisation $\underline{\omega }\in \Omega$, the position of the particles is given by $f^n_{\underline{\omega }}(\boldsymbol{x}^1)$ and $f^n_{\underline{\omega }}(\boldsymbol{x}^2)$ respectively, where there holds as well $f^n_{\underline{\omega }}(\boldsymbol{x}^1)\ne f^n_{\underline{\omega }}(\boldsymbol{x}^2)$.

Precisely to hold account of such points in the diagonal we introduce the notation

$$ \Delta = \{(\boldsymbol{x}^1,\boldsymbol{x}^2)\in \mathbb {T}^3\times \mathbb {T}^3\mid \boldsymbol{x}^1=\boldsymbol{x}^2\}\subset \mathbb {T}^3\times \mathbb {T}^3. $$

The necessity of removing this subset comes from the fact that there is a invariant measure supported in Δ, namely the complement set of the diagonal is not reachable from the diagonal. Therefore exponential ergodicity in the full space $\mathbb {T}^3\times \mathbb {T}^3$ is not viable. In particular, the dynamics in Δ are perfectly described by the one-point chain and well-posedness of the ODE

$$ \frac{\textrm{d}}{\textrm{d}t}\phi _t(\boldsymbol{x}) = u(t,\phi _t(\boldsymbol{x})), \quad t>0,\boldsymbol{x}\in \mathbb {T}^3 $$

with a Lipschitz vector field *u* like (1.8) yields that the neither the diagonal is reachable from out of the diagonal, giving really (at least) two different invariant measures in $\mathbb {T}^3\times \mathbb {T}^3$.

With this notation in hand, for any Borel set $A^{(2)}\in \mathcal {B}((\mathbb {T}^3\times \mathbb {T}^3)\setminus \Delta )$, and any $(\boldsymbol{x}^1,\boldsymbol{x}^2)\in (\mathbb {T}^3\times \mathbb {T}^3)\setminus \Delta$, we define the two-point Markov chain via

3.6 $$\begin{aligned} P^{(2)}((\boldsymbol{x}^1,\boldsymbol{x}^2),A^{(2)})= \mathbb {P}_0\left[ \left( f_\omega (\boldsymbol{x}^1),f_\omega (\boldsymbol{x}^2)\right) \in A^{(2)} \right] . \end{aligned}$$

Just like for the projective chain, let us introduce the following short notation,

$$ \boldsymbol{x}^1_n = f_{\omega _n}(\boldsymbol{x}^1_{n-1}) \quad \text {and}\quad \boldsymbol{x}^2_n = f_{\omega _n}(\boldsymbol{x}^2_{n-1}) $$

where $\boldsymbol{x}^1_0 = \boldsymbol{x}^1$ and $\boldsymbol{x}^2_0 = \boldsymbol{x}^2$. Analogously to the previous section, to prove that the two-point chain is topologically irreducible, we will show that it is indeed exactly controllable.

##### Proposition 3.6

Given any two elements $(\boldsymbol{x}^1,\boldsymbol{x}^2),(\boldsymbol{x}^{\star ,1},\boldsymbol{x}^{\star ,2})\in (\mathbb {T}^3\times \mathbb {T}^3)\setminus \Delta$, there exist $N\in \mathbb {N}$ and $\underline{\omega }^N=(\omega _1,\ldots ,\omega _N)\in \Omega _0^N$ such that

$$ f_{\underline{\omega }^N}(\boldsymbol{x}^1)=\boldsymbol{x}^{\star ,1} \quad \text {and} \quad f_{\underline{\omega }^N}(\boldsymbol{x}^2)=\boldsymbol{x}^{\star ,2}. $$

The proof of this proposition is split in a number of steps. The strategy consists on selecting appropriate noise realisations so that we can bring the two particles arbitrarily close, then we can bring one of them to a target $\overline{\boldsymbol{x}}$ via rigid rotations, so that the distance between both particles remains fixed. Finally, we close the argument using the invertibility of the vector field.

##### Lemma 3.7

Let $(\boldsymbol{x}^1,\boldsymbol{x}^2)\in (\mathbb {T}^3\times \mathbb {T}^3)\setminus \Delta$. Given any ε>0, there exists $N_1\in \mathbb {N}$ and $\underline{\omega }^{N_1}\in \Omega _0^{N_1}$ such that $x^1_{N_1}=x^2_{N_1}$, $y^1_{N_1}=y^2_{N_1}$, and $|z^1_{N_1}-z^2_{N_1}|<\varepsilon$. In particular,

$$ \text {dist}_{\mathbb {T}^3}(\boldsymbol{x}^1_{N_1},\boldsymbol{x}^2_{N_1}) <\varepsilon . $$

##### Proof

Let ε>0 be fixed, and assume that $|z_0^1-z_0^2|\ge \varepsilon$. First of all let us choose $\textsf{B}=\textsf{C}=0$, then notice that we can write the distance between the two points as

$$\begin{aligned} x_1^1-x_1^2&= x_0^1-x_0^2 + \textsf{A}(\sin (z_0^1+\alpha )-\sin (z_0^1+\alpha )) \\&= x_0^1-x_0^2 + 2\textsf{A}\sin \left( \frac{z^1_0-z^2_0}{2}\right) \cos \left( \frac{z^1_0+z^2_0}{2} + \alpha \right) ,\\ y_1^1-y_1^2&= y_0^1-y_0^2 + \textsf{A}(\cos (z_0^1+\alpha )-\cos (z_0^1+\alpha )) \\&= y_0^1-y_0^2 + 2\textsf{A}\sin \left( \frac{z^1_0-z^2_0}{2}\right) \sin \left( \frac{z^1_0+z^2_0}{2} + \alpha \right) , \end{aligned}$$

and $z_0^1-z_0^2=z_1^1-z_1^2$. Now we select α such that

$$ \cos \left( \frac{z^1_0+z^2_0}{2} + \alpha \right) = {0}, $$

namely, $x_0^1-x_0^2=x_1^1-x_1^2$ is a fixed point as well. The idea is to choose $\textsf{A}$ such that $y_0^1-y_0^2=\varepsilon$, however this might not be possible in one iteration because |A|≤U. Nonetheless, we can define a finite sequence $\{\textsf{A}_n\}_{1\le n\le M_1}\subset [-U,U]$ for some $M_1\in \mathbb {N}$, and

$$ x_n^1-x_n^2 = x_{n-1}^1-x_{n-1}^2 + 2\textsf{A}_n\sin \left( \frac{z^1_0-z^2_0}{2}\right) , $$

such that there holds $x_{M_1}^1-x_{M_1}^2=\varepsilon$. In a similar fashion, we find that there exists a some natural number $M_2$, so that choosing now $\alpha _n$ such that

$$ \cos \left( \frac{z^1_0+z^2_0}{2} + \alpha _n\right) = 0, $$

for all $M_1+1\le n\le M_2$, we find another finite sequence $\{\textsf{A}_n\}_{M_1+1\le n\le M_2}\subset [-U,U]$ such that $y_{M_2}^1-y_{M_2}^2=\varepsilon$ and $x_{M_2}^1-x_{M_2}^2=\varepsilon$. Furthermore, by now choosing $\textsf{A}=\textsf{B}=0$ and $\textsf{C}_n$ and $\gamma _n$ appropriately, we can repeat this argument to bring $z^1_n{-}z^2_n$ as well to distance ε in $M_3{+}M_2{+}M_1\in \mathbb {N}$ iterations, so that

$$ x_{M_3}^1-x_{M_3}^2=y_{M_3}^1-y_{M_3}^2=z_{M_3}^1-z_{M_3}^2=\varepsilon . $$

Finally, selecting again $\textsf{B}_{M_3+1}={\textsf{C}}_{M_3+1}=0$, and $\alpha _{M_3+1}$ so that $y_{M_3+1}^1-{y_{M_3+1}^2}=y_{M_3}^1-{y_{M_3}^2}$, we find that we can choose $\textsf{A}_{M_3+1}$ such that

$$ 0 = x_{M_3+1}^1-x_{M_3+1} = \varepsilon + 2\textsf{A}_{M_3+1}\sin \left( \frac{\varepsilon }{2}\right) , $$

since

$$ \textsf{A}_{M_3+1} = -\frac{\varepsilon }{2\sin (\varepsilon /2)} \in [-U,U] $$

provided that U>1 and ε is small enough. In particular, the choice U=π yields an upper bound for the admissible ε>0. To sum up, we apply one final iteration with $\alpha _{M_3+2} = \alpha _{M_3+1}+\pi /2$ and the same choice of $\textsf{A}_{M_3+2}=\textsf{A}_{M_3+1}$, so that $y_{M_3+2}^1-y^2_{M_3+2}=0$, and thus there concludes the proof of the lemma. □

##### Lemma 3.8

Let $(\boldsymbol{x}^1,\boldsymbol{x}^2)\in (\mathbb {T}^3\times \mathbb {T}^3)\setminus \Delta$ be such that $x^1-x^2=0$ and $y^1-y^2=0$. Given any $\overline{\boldsymbol{x}}\in \mathbb {T}^3$, there exist $N_2\in \mathbb {N}$ and $\underline{\omega }^{N_2}\in \Omega _0^{N_2}$ so that

$$ {\boldsymbol{x}^1_{N_2}=\overline{\boldsymbol{x}}} \qquad \text {and} \qquad \text {dist}_{\mathbb {T}^3}(\overline{\boldsymbol{x}},\boldsymbol{x}^2_{N_2})=\text {dist}_{\mathbb {T}^3}(\boldsymbol{x}^1,\boldsymbol{x}^2). $$

##### Proof

The proof of this lemma mimics the proof of Lemma 3.4. On the one hand, notice that if $x^1-x^2=0$ and $y^1-y^2=0$, then it suffices to impose $\textsf{A}_n=0$ in order to make sure that $\boldsymbol{x}^1_n-\boldsymbol{x}^2_n=\boldsymbol{x}^1-\boldsymbol{x}^2$ for all *n*. Indeed,

3.7 $$\begin{aligned} {[}f_{(\textsf{A},\alpha )}(\boldsymbol{x}^1)]_1-[f_{(\textsf{A},\alpha )}(\boldsymbol{x}^2)]_1&= x^1-x^2 + 2\textsf{A}\sin \left( \frac{z^1-z^2}{2}\right) \cos \left( \frac{z^1+z^2}{2} + \alpha \right) ,\end{aligned}$$

3.8 $$\begin{aligned} {[}f_{(\textsf{A},\alpha )}(\boldsymbol{x}^1)]_2-[f_{(\textsf{A},\alpha )}(\boldsymbol{x}^2)]_2&= y^1-y^2 + 2\textsf{A}\sin \left( \frac{z^1-z^2}{2}\right) \sin \left( \frac{z^1+z^2}{2} + \alpha \right) ,\end{aligned}$$

3.9 $$\begin{aligned} {[}f_{(\textsf{A},\alpha )}(\boldsymbol{x}^1)]_3-[f_{(\textsf{A},\alpha )}(\boldsymbol{x}^2)]_3&= z^1-z^2, \end{aligned}$$

so $\textsf{A}=0$ implies $f_{(\textsf{A},\alpha )}(\boldsymbol{x}^1)-f_{(\textsf{A},\alpha )}(\boldsymbol{x}^2) = \boldsymbol{x}^1-\boldsymbol{x}^2$. Moreover, the $(\textsf{B},\beta )-$flow and the $(\textsf{C},\gamma )-$flow leave the vector $\boldsymbol{x}^1 - \boldsymbol{x}^2$ invariant regardless of the choice of $\textsf{B}$ and $\textsf{C}$ due to the fact that $x^1-x^2=0$ and $y^1-y^2=0$. Hence, $\textsf{A}=0$ yields

$$ f_\omega (\boldsymbol{x}^1)-f_\omega (\boldsymbol{x}^2) = \left( f_{(\textsf{C},\gamma )}\circ f_{(\textsf{B},\beta )}\circ f_{(\textsf{A},\alpha )} \right) (\boldsymbol{x}^1) - \left( f_{(\textsf{C},\gamma )}\circ f_{(\textsf{B},\beta )}\circ f_{(\textsf{A},\alpha )} \right) (\boldsymbol{x}^2) = \boldsymbol{x}^1 - \boldsymbol{x}^2. $$

Now the goal is to show that we can reach $\overline{\boldsymbol{x}}$ from $\boldsymbol{x}^1$ in $N_2$ iterations of the flow, under the restriction $\textsf{A}_n=0$ for all $n\le N_2$. To do so we only need to follow the proof of Lemma 3.4 but imposing $\textsf{A}=0$ instead of $\textsf{B}=0$. Namely we choose $\textsf{B},\textsf{C}\in [-U,U]$ and $\beta ,\gamma \in [0,2\pi )$ so that

$$ \begin{pmatrix} x_1^1\\ y_1^1\\ z_1^1 \end{pmatrix} = \begin{pmatrix} x^1 + \textsf{C}\cos (\gamma + y^1)\\ y^1 + \textsf{B}\sin (\beta + x^1) \\ z^1 + \textsf{B}\cos (\beta + x^1) + \textsf{C}\sin (\gamma + y^1) \end{pmatrix} $$

is equal to $\overline{\boldsymbol{x}}$. This might be not possible in one step, but it is clear that there exists $N_2\in \mathbb {N}$ such that after $N_2$ iterations of the flow we arrive to $\boldsymbol{x}^1 = \overline{\boldsymbol{x}}$. This concludes the proof of the lemma. □

##### Proof of Proposition 3.6

Fix $(\boldsymbol{x}^1,\boldsymbol{x}^2),(\boldsymbol{x}^{\star ,1},\boldsymbol{x}^{\star ,2})\in (\mathbb {T}^3\times \mathbb {T}^3)\setminus \Delta$ and fix ε>0. On the one hand we can apply Lemma 3.7 to the inverse of the flow map $f^{-1}_\omega$ (see Remark 3.5), to deduce that there exist $N_1\in \mathbb {N}$ and $\underline{\omega }^{N_1}\in \Omega _0^{N_1}$ such that

$$ \overline{x}^1 = \overline{x}^2, \quad \overline{y}^1 = \overline{y}^2 \quad \text {and} \quad \text {dist}_{\mathbb {T}^3}(\overline{z}^1,\overline{z}^2)=\varepsilon _0, $$

where $\overline{\boldsymbol{x}}^1 = f_{\underline{\omega }^{N_1}}^{-1}(\boldsymbol{x}^{\star ,1})$ and $\overline{\boldsymbol{x}}^2 = f_{\underline{\omega }^{N_1}}^{-1}(\boldsymbol{x}^{\star ,2})$. On the other hand, Lemma 3.7 applied to the (forward) flow map starting from $(\boldsymbol{x}^1,\boldsymbol{x}^2)$ yields that we can find $N_2\in \mathbb {N}$ and $\underline{\omega }^{N_2}\in \Omega _0^{N_2}$ such that

$$ [f_{\underline{\omega }^{N_2}}(\boldsymbol{x}^1)]_1 = [f_{\underline{\omega }^{N_2}}(\boldsymbol{x}^2)]_1, \quad [f_{\underline{\omega }^{N_2}}(\boldsymbol{x}^1)]_2 = [f_{\underline{\omega }^{N_2}}(\boldsymbol{x}^2)]_2, $$

$$ [f_{\underline{\omega }^{N_2}}(\boldsymbol{x}^1)]_3 - [f_{\underline{\omega }^{N_2}}(\boldsymbol{x}^2)]_3=\varepsilon . $$

Now, Lemma 3.8 ensures that there exist $N_3\in \mathbb {N}$ and $\underline{\omega }^{N_2}\in \Omega _0^{N_3}$ such that

$$ f_{\underline{\omega }^{N_3}}( f_{\underline{\omega }^{N_2}}(\boldsymbol{x}^1)) = \overline{\boldsymbol{x}}^1 \quad \text {and} \quad f_{\underline{\omega }^{N_3}}( f_{\underline{\omega }^{N_2}}(\boldsymbol{x}^1) )- f_{\underline{\omega }^{N_3}}( f_{\underline{\omega }^{N_2}}(\boldsymbol{x}^2) = \begin{pmatrix} 0 \\ 0 \\ \varepsilon \end{pmatrix} . $$

To sum up, we define $N=N_2+N_3+N_1$ and the noise vector $\underline{\omega }^N=(\underline{\omega }^{N_2},\underline{\omega }^{N_3},\underline{\omega }^{N_1})\in \Omega _0^N$, then we found that

$$ f_{\underline{\omega }^{N}}( \boldsymbol{x}^1 )= \boldsymbol{x}^{\star ,1} \quad \text {and} \quad f_{\underline{\omega }^{N}}( \boldsymbol{x}^2 )= \boldsymbol{x}^{\star ,2}, $$

and the statement of the proposition follows. □

Regarding the existence of a small set for the two point process, we go back to Lemma 2.6 yet again. Given any $n\in \mathbb {N}$ and $(\boldsymbol{x}^1,\boldsymbol{x}^2)\in (\mathbb {T}^3\times \mathbb {T}^3)\setminus \Delta$, we define the mapping

$$\begin{aligned} \Phi ^{(2)}_{(\boldsymbol{x}^1,\boldsymbol{x}^2)} : \Omega _0^n&\rightarrow (\mathbb {T}^3\times \mathbb {T}^3)\setminus \Delta \\ \underline{\omega }^n \  &\mapsto \ \Phi ^{(2)}_{(\boldsymbol{x}^1,\boldsymbol{x}^2)} (\underline{\omega }^n) = (f_{\underline{\omega }^n}(\boldsymbol{x}^1),f_{\underline{\omega }^n}(\boldsymbol{x}^2)). \end{aligned}$$

Hence, by Lemma 2.6, as long as we can find a choice of $n\in \mathbb {N}$, $(\boldsymbol{x}^1,\boldsymbol{x}^2)\in (\mathbb {T}^3\times \mathbb {T}^3)\setminus \Delta$ and $\underline{\omega }^n\in \Omega _0^n$ for which $D_{\underline{\omega }^n}\Phi ^{(2)}_{(\boldsymbol{x}^1,\boldsymbol{x}^2)}$ has rank equal to $\dim (\mathbb {T}^3\times \mathbb {T}^3)=6$, the two-point Markov chain $P^{(2)}$ will admit a small set. Notice that since on each time step the noise vector $\omega =(\textsf{A},\textsf{B},\textsf{C},\alpha ,\beta ,\gamma )\in \Omega _0$ is six dimensional, we can restrict ourselves to the case n=1. In particular we choose

$$ \boldsymbol{x}^1=(0,0,0),\qquad \boldsymbol{x}^2=\left( \frac{\pi }{2},\frac{\pi }{2},\frac{\pi }{2} \right) , \qquad (\textsf{A},\textsf{B},\textsf{C})=(\pi ,\pi ,1), \qquad (\alpha ,\beta ,\gamma )=(0,0,0), $$

so that there yields the matrix

$$ D_{\omega }\Phi ^{(2)}_{(\boldsymbol{x}^1,\boldsymbol{x}^2)} = \begin{pmatrix} 0 &  0 &  -1 &  \pi &  0 &  0\\ 1 &  0 &  0 &  \pi ^2 &  \pi &  0\\ -1 &  1 &  0 &  -\pi ^2 &  -\pi &  -1\\ 1 &  -1 &  0 &  -\pi &  0 &  1\\ 0 &  -1 &  0 &  -\pi &  0 &  0\\ \pi &  0 &  -1 &  0 &  \pi &  0 \end{pmatrix} $$

which has determinant equal to $2\pi ^2$, and thus full rank.

Unlike for the projective and on-point chains, irreducibility + aperiodicity + small sets does not imply geometric ergodicity since the domain $(\mathbb {T}^3\times \mathbb {T}^3)\setminus \Delta$ is not compact. The Lyapunov-drift condition from Theorem 3 is also needed, and in order to to prove that it is also satisfied we need first to deal with the top Lyapunov exponent and Lemma 2.9.

### Top Lyapunov exponent

The final ingredient missing for the main theorem is the positivity of the top Lyapunov exponent for the continuous random dynamical system $f_{\underline{\omega }}^n$ on $\mathbb {T}^3$.

In order to do so we will apply Proposition 2.10, that give sufficient conditions to rule out the case $\lambda _1=0$ via Furstenberg’s criterion, i.e. Proposition 2.4. Observe that conditions (L1) and (L2) in Proposition 2.10 coincide with the conditions required for the small set from Proposition 2.6, and we have already found $n\in \mathbb {N}$, $\underline{\omega }_\star ^n\in \Omega _0^n$ and $\boldsymbol{x}^\star \in \mathbb {T}^3$ so that this holds for the one-point process in Section 3.1.1. However, to prove that $f_{\underline{\omega }}^n$ has a positive tope Lyapunov exponent we must come up with a choice of $n\in \mathbb {N}$, $\underline{\omega }_\star ^n\in \Omega _0^n$ and $\boldsymbol{x}^\star \in \mathbb {T}^3$ so that the three conditions (L1)–(L3) are satisfied simultaneously.

Notice that $\dim SL_3(\mathbb {R})=8$, since we need $\Phi _{\boldsymbol{x}^\star }(\underline{\omega }^n)$ to be a submersion we find that

$$ \dim \ker D_{\underline{\omega }^n_\star }\Phi _{\boldsymbol{x}}\le 6n-3. $$

Therefore we must choose n≥2 in order to be able to find parameters for which $D_{\underline{\omega }^n_\star }\Psi _{\boldsymbol{x}^\star }$ restricted to $\ker D_{\underline{\omega }^n_\star }\Phi _{\boldsymbol{x}^\star }$ is a surjection onto $T_{\Psi _{\boldsymbol{x}^\star }(\underline{\omega }^n)}SL_3(\mathbb {R})$.

We choose n=2 and make the following selection of parameters

$$ \boldsymbol{x}^\star =(0,0,0),\quad (\textsf{A}_1,\textsf{B}_1,\textsf{C}_1) = (\pi ,\pi ,\pi ),\quad (\textsf{A}_2,\textsf{B}_2,\textsf{C}_2)=(\pi ,1,0), $$

and

$$ (\alpha _1,\beta _1,\gamma _1) = \left( \frac{\pi }{2}, \frac{\pi }{2},\frac{\pi }{2}\right) ,\quad (\alpha _2,\beta _2,\gamma _2) = (0,0,0). $$

This particular set of parameters was selected because it produces matrices that are the most structured and readable among all configurations we found satisfying the properties required for Proposition 2.10. Since the computations at this stage are lengthy and tedious, we present a schematic version of the calculations for the reader’s convenience. We set $\underline{\omega }_\star ^2=(\omega _1,\omega _2)$ with $\omega _1=(\textsf{A}_1,\textsf{B}_1,\textsf{C}_1,\alpha _1,\beta _1,\gamma _1)$ and $\omega _2=(\textsf{A}_2,\textsf{B}_2,\textsf{C}_2,\alpha _2,\beta _2,\gamma _2)$, so first of all we obtain the matrix

**Figure:**
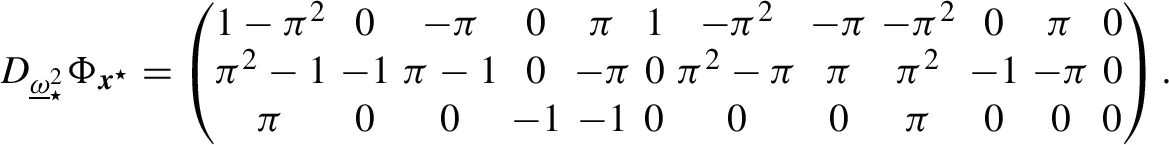

Since the rank of $D_{\underline{\omega }_\star ^2}\Phi _{\boldsymbol{x}^\star }$ is 3, we see that this choice of parameters verifies property (2) from Proposition 2.10. Moreover, property (1) is also verified by the definition of the noise in Section 1.3, and therefore there is only property (3) left to be checked.

To verify condition (3) from Proposition 2.10, we explicitly compute the matrix $\mathcal {A}=D_{\underline{\omega }_2^\star }\Psi _{\boldsymbol{x}^\star }$ and construct a specially chosen basis $\mathcal {K}$ for the kernel of the matrix $D_{\underline{\omega }_\star ^2}\Phi _{\boldsymbol{x}^\star }$. We then show that the restriction of $\mathcal {A}$ to this kernel, represented by the matrix product $\mathcal {A}\mathcal {K}$, thereby demonstrating surjectivity onto $T_{\Psi _{\boldsymbol{x}^\star }(\underline{\omega }_2^\star )}SL_3(\mathbb {R})$. First of all, observe that the kernel of $D_{\underline{\omega }_\star ^2}\Phi _{\boldsymbol{x}^\star }$ is 9 dimensional, and it is spanned by the columns of the following matrix

$$ \mathcal {K} = \begin{pmatrix} 0 &  0 &  0 &  -1 &  0 &  0 &  0 &  \pi ^{-1} &  \pi ^{-1} \\ 0 &  -1 &  -1 &  \pi ^{-1} &  1 &  0 &  1-\pi ^{-1} &  -\pi ^{-2} &  1-\pi ^{-2} \\ 0 &  1 &  0 &  -\pi ^{-1} &  -1 &  -\pi &  \pi ^{-1} &  \pi ^{-2} &  -1+\pi ^{-2} \\ 0 &  0 &  0 &  0 &  0 &  0 &  0 &  0 &  1 \\ 0 &  0 &  0 &  0 &  0 &  0 &  0 &  1 &  0 \\ 0 &  0 &  0 &  0 &  0 &  0 &  1 &  0 &  0 \\ 0 &  0 &  0 &  0 &  0 &  1 &  0 &  0 &  0 \\ 0 &  0 &  0 &  0 &  1 &  0 &  0 &  0 &  0 \\ 0 &  0 &  0 &  1 &  0 &  0 &  0 &  0 &  0 \\ 0 &  0 &  1 &  0 &  0 &  0 &  0 &  0 &  0 \\ 0 &  1 &  0 &  0 &  0 &  0 &  0 &  0 &  0 \\ 1 &  0 &  0 &  0 &  0 &  0 &  0 &  0 &  0 \\ \end{pmatrix} $$

Next, we identify the space of 3×3 real matrices with $\mathbb {R}^9$ via the standard parametrisation,

$$ (a_{ij})_{i,j=1}^3 \mapsto (a_{11},a_{12},a_{13},a_{21},a_{22},a_{23},a_{31},a_{32},a_{33})^\top . $$

By means of this parametrisation, we introduce $\mathcal {A}=D_{\underline{\omega }_\star ^2}\Psi _{\boldsymbol{x}^\star }$. Due to space limitations in this format, we decide to split the 9×12 matrix $\mathcal {A}$ in two 9×6 submatrices, namely $\mathcal {A}=(\mathcal {A}_1|\mathcal {A}_2)$. For our choice of parameters we find that

$$ \mathcal {A}_1 = \begin{pmatrix} \pi ^2 &  -\pi &  -\pi &  0 &  0 &  0 \\ 0 &  0 &  \pi ^2 &  0 &  1 &  0 \\ -\pi &  -1 &  -\pi ^3 &  0 &  -\pi &  0 \\ \pi ^3-\pi ^2+\pi &  \pi &  \pi &  \pi ^2-1 &  -\pi ^2 &  0 \\ 0 &  0 &  -\pi ^2 &  -\pi &  -1 &  0 \\ \pi ^2+\pi -1 &  1 &  \pi ^3 &  \pi ^2+\pi &  0 &  0 \\ (\pi ^2-1)^2 &  0 &  \pi ^3-\pi +1 &  0 &  -\pi ^3+\pi &  \pi ^2-1 \\ -\pi ^3+\pi &  0 &  -\pi ^2-\pi &  0 &  \pi ^2 &  -\pi +1 \\ \pi (\pi +1)^2(\pi -1) &  0 &  \pi ^2(\pi +2) &  0 &  -\pi ^2(\pi +1) &  \pi ^2 \end{pmatrix}, $$

and

$$ \mathcal {A}_2 = \begin{pmatrix} 0 &  0 &  \pi ^2 &  0 &  0 &  0 \\ \pi ^3 &  0 &  0 &  0 &  -\pi ^2 &  0 \\ -\pi ^4+\pi ^3-\pi &  0 &  0 &  0 &  \pi ^3 &  0 \\ 0 &  \pi ^2 &  \pi ^3-\pi ^2+\pi &  0 &  0 &  0 \\ -\pi ^3 &  0 &  0 &  0 &  \pi ^2 &  0 \\ \pi ^4-\pi ^3+\pi &  \pi &  \pi ^2 &  0 &  -\pi ^3 &  0 \\ \pi ^2(\pi ^2-1) &  \pi ^3-\pi &  \pi ^2(\pi ^2-1) &  -\pi ^2+1 &  -\pi ^3+\pi &  0 \\ -\pi ^3-\pi ^2 &  -\pi ^2 &  -\pi ^3 &  \pi &  \pi ^2+\pi &  0 \\ \pi ^4+2\pi ^3-\pi ^2 &  \pi ^3+\pi ^2 &  \pi ^4+\pi ^3 &  -\pi ^2-\pi &  -\pi ^3-2\pi ^2 &  0 \\ \end{pmatrix}. $$

Hence, to sum up we need to show that $\mathcal {A}=(\mathcal {A}_1|\mathcal {A}_2)$ restricted to $\ker (D_{\underline{\omega }^n_\star }\Phi _{\boldsymbol{x}})$ as a linear operator has rank ≥8. A straightforward computation yields that $\mathcal {A}\mathcal {K}$ is a 9×9 matrix that we present here split in two as before $\mathcal {A}\mathcal {K}=((\mathcal {A}\mathcal {K})_1|(\mathcal {A}\mathcal {K})_2)$, where $(\mathcal {A}\mathcal {K})_1$ is a 9×5 matrix, and $(\mathcal {A}\mathcal {K})_2$ is a 9×4 matrix with the following form

$$ (\mathcal {A}\mathcal {K})_1 = \begin{pmatrix} 0 &  0 &  \pi &  0 &  0 \\ 0 &  0 &  0 &  -\pi ^2 &  -\pi ^2 \\ 0 &  1 &  1 &  \pi ^2+\pi -\pi ^{-1} &  \pi ^3-1 \\ 0 &  0 &  -\pi &  0 &  \pi ^2 \\ 0 &  0 &  0 &  \pi &  \pi ^2 \\ 0 &  -1 &  -1 &  \pi ^{-1}(\pi -1)(\pi +1)^2 &  -\pi ^3+\pi +1 \\ 0 &  1 &  1-\pi ^2 &  -\pi ^{-1} &  -1 \\ 0 &  0 &  \pi &  1 &  \pi \\ 0 &  0 &  -\pi ^2-\pi &  -\pi &  -\pi ^2 \end{pmatrix}, $$

and

$$ (\mathcal {A}\mathcal {K})_2 = \begin{pmatrix} \pi ^2 &  -\pi &  \pi &  \pi \\ 0 &  \pi &  2 &  -\pi ^2+1\\ \pi ^3-\pi &  -\pi ^2-1+\pi ^{-1} &  -2\pi -1+\pi ^{-2} &  \pi ^3-\pi -2+\pi ^{-2}\\ -\pi ^2 &  \pi &  -\pi +1 &  2\pi ^2-\pi \\ 0 &  -\pi &  -2 &  \pi ^2-\pi -1\\ -\pi ^3+\pi &  \pi ^2+1-\pi ^{-1} &  2\pi +1-\pi ^{-1}-\pi ^{-2} &  -\pi ^3+\pi ^2+3\pi +2-\pi ^{-1}-\pi ^{-2}\\ -\pi &  2\pi ^2-2+\pi ^{-1} &  \pi ^{-2} &  -1+\pi ^{-2}\\ 0 &  -2\pi &  -\pi ^{-1} &  \pi -\pi ^{-1}\\ -\pi ^2 &  2\pi (\pi +1) &  1 &  -\pi ^2+1 \end{pmatrix}. $$

The matrix $\mathcal {A}\mathcal {K}$ has rank 8 since all columns are linearly independent, except the first trivial column in $(\mathcal {A}\mathcal {K})_1$. This completes the requirements needed to show, via Proposition 2.10, that the top Lyapunov exponent is strictly positive.

#### Proof of Theorem 1

We finally have all the bits needed to close the proof of Theorem 1. By means of Proposition 2.5 we see that the goal is to show that the two-point chain $P^{(2)}$ is geometrically ergodic. To begin with notice that the basic assumptions (H1)–(H3) are trivially satisfied by the random ABC vector field (1.8) with absolutely continuous noise. In Sections 3.1.1 and 3.1.2 we prove geometric ergodicity of the one-point and projective chains respectively. Additionally in Section 3.1.3 we demonstrate that the two-point chain satisfies the first three requirements from Harris theorem, namely irreducibility, aperiodicity and small sets. Finally in Section 3.2 we show that the top Lyapunov exponent is strictly positive for almost all $\boldsymbol{x}\in \mathbb {T}^3$ and $\underline{\omega }\in \Omega$, which yields via Lemma 2.9 that the two-poin chain $P^{(2)}$ satisfies the Lyapunov-drift condition. $P^{(2)}$ is geometrically ergodic, and hence the claim in Theorem 1 follows for all discrete times $t=n\in \mathbb {N}$. To include all the positive real times we write t=n+τ>0 with $n\in \mathbb {N}$ and $\tau \in [0,1)$, then via Proposition 2.5 we obtain the estimate

$$ \left| \int _{\mathbb {T}^3} g(\boldsymbol{x})h(\phi _{n+\tau }^\kappa (\boldsymbol{x}))\textrm{d}\boldsymbol{x}\right| \le \widehat{C}_{\underline{\omega },\kappa }\Vert g\Vert _{H^s} \Vert h\circ \phi _\tau ^\kappa \Vert _{H^s}\textrm{e}^{-\lambda _s n}. $$

We claim that we can control the $H^s$ norm of $h\circ \phi _\tau ^\kappa$ by the $H^s$ norm of *h*. To see this, we notice that an application of the chain rule yields that all integer derivatives of the flow map $\phi _\tau ^\kappa$ are bounded. Indeed, we have $\Vert \nabla \phi _\tau ^\kappa \Vert _{L^\infty } \le \textrm{e}^{\Vert \nabla u\Vert _{L^\infty }}$ for all $0\le \tau \le 1$, and almost every realization of the noise induced by the diffusion. Then, for any $m\in \mathbb {N}$, via an iterative argument and Sobolev embeddings, we use the bounds on $\Vert \nabla ^{m-1}\phi _\tau ^\kappa \Vert _{L^\infty }$ to find an estimate for $\Vert \nabla ^m\phi _\tau ^\kappa \Vert _{L^\infty }$. All in all, we obtain that for any $m\in \mathbb {N}$ there exists $C_{m,u}>0$ depending only on $\Vert \nabla ^mu\Vert _{L^\infty }$ such that $\Vert \nabla ^m\phi _\tau ^\kappa \Vert _{L^\infty } \le C_{m,u}$. Another application of the chain rule for $h\circ \phi _\tau ^\kappa$ yields that $\Vert h\circ \phi _\tau ^\kappa \Vert _{H^m} \lesssim _{m,u}\Vert h\Vert _{H^m}$, where the constant absorbed in $\lesssim _{m,u}$ only depends on $\Vert \nabla ^mu\Vert _{L^\infty }$. To cover all real exponents s>0 we can use the Slobodeckij seminorm, see e.g. [34]

$$ \Vert f\Vert _{H^s}^2 \simeq \sum _{|\alpha |=\lfloor s\rfloor }\iint _{\mathbb {T}^3\times \mathbb {T}^3} \frac{|\partial ^\alpha f(\boldsymbol{x})-\partial ^\alpha f(\boldsymbol{y})|^2}{|\boldsymbol{x}-\boldsymbol{y}|^{2s+3}} \textrm{d}\boldsymbol{x}\textrm{d}\boldsymbol{y}$$

where $\lfloor s\rfloor \in \mathbb {N}$ denotes the largest natural number that is smaller or equal to s>0. Indeed, making use of the estimates for integer indices together with Sobolev embeddings in $\mathbb {T}^3$, we find that

$$ \Vert h\circ \phi _\tau ^\kappa \Vert _{H^s}^2 \lesssim \Vert \nabla \phi _\tau ^\kappa \Vert _{L^\infty }^{2s+3} \sum _{|\alpha |=\lfloor s\rfloor }\iint _{\mathbb {T}^3\times \mathbb {T}^3} \frac{|\partial ^\alpha (h\circ \phi _\tau ^\kappa )(\boldsymbol{x})-\partial ^\alpha (h\circ \phi _\tau ^\kappa )(\boldsymbol{y})|^2}{|\phi _\tau ^\kappa (\boldsymbol{x})-\phi _\tau ^\kappa (\boldsymbol{y})|^{2s+3}} \textrm{d}\boldsymbol{x}\textrm{d}\boldsymbol{y}\lesssim _{s,u}\Vert h\Vert _{H^s}^2, $$

and the statement of Theorem 1 follows directly from here. □

## Ideal kinematic dynamo: proof of Theorem 2

The last part of this paper is devoted to the ideal dynamo problem. The results presented in the previous sections are directly applicable to obtain the growth of a magnetic field that is advected and stretched by the random ABC vector field (1.8) via the ideal kinematic dynamo equation,

$$ \partial _t B + (u\cdot \nabla ) B - (B\cdot \nabla ) u = 0, \quad \nabla \cdot B = 0, $$

with t>0, $\boldsymbol{x}\in \mathbb {T}^3$, and with initial datum $B(0,\cdot ) = B_0\in L^1(\mathbb {T}^3)$.

When κ=0, solutions to (1.4) can be recovered from the flow map solving the ODE

4.1 $$\begin{aligned} {\frac{\textrm{d}}{\textrm{d}t}}\phi _t(\boldsymbol{x}) = u(t,\phi _t(\boldsymbol{x})),\quad \phi _0(\boldsymbol{x}) = \boldsymbol{x}, \end{aligned}$$

via the formula (compare with (1.7))

4.2 $$\begin{aligned} B(t,\phi _t(\boldsymbol{x})) = D_{\boldsymbol{x}}\phi _t B_0(\boldsymbol{x}). \end{aligned}$$

Indeed, writing (4.2) component-wise and taking a time derivative, for i=1,2,3 we find that

$$\begin{aligned} (\partial _t B^i +u\cdot \nabla B^i)(t,\phi _t(\boldsymbol{x}))&={\frac{\textrm{d}}{\textrm{d}t}}B^i(t,\phi _t(\boldsymbol{x})) ={\frac{\textrm{d}}{\textrm{d}t}}\left( \partial _j \phi _t^i(\boldsymbol{x})B_0^j(\boldsymbol{x})\right) =\partial _j \left( u^i(t,\phi _t(\boldsymbol{x}))\right) B_0^j(\boldsymbol{x})\\&=\nabla u^i(t,\phi _t(\boldsymbol{x})) \cdot \partial _j\phi _t(\boldsymbol{x}) B_0^j(\boldsymbol{x}) =\partial _j \phi ^\ell (\boldsymbol{x}) B_0^j(\boldsymbol{x}) \partial _\ell u^i (t,\phi _t(\boldsymbol{x}))\\&=(B^\ell \cdot \partial _\ell u^i)(t,\phi _t(\boldsymbol{x})) =(B\cdot \nabla u^i)(t,\phi _t(\boldsymbol{x})), \end{aligned}$$

as needed. In order to show that the ABC flow with random coefficients and random phases is an ideal kinematic dynamo, we will use two key features that we introduced for the mixing problem: the positivity of the top Lyapunov exponent, and the ergodicity of the projective process. This argument is inspired by the ideas used in [9] to prove growth of the gradient of the passive scalar in every $L^p$ space.

### Proposition 4.1

Assume that the projective Markov transition kernel $\hat{P}$ has a unique stationary measure. Then for a.e. $\boldsymbol{x}\in \mathbb {T}^3$, and all $\boldsymbol{v}\in \mathbb {S}^2$ we find that

$$ \lim _{n\rightarrow \infty }\frac{1}{n}\log |D_{\boldsymbol{x}} f_{\underline{\omega }}^n\boldsymbol{v}| = \lambda _1 $$

is satisfied for $\mathbb {P}-$almost all $\underline{\omega }\in \Omega$.

This proposition, states that for almost every realization, the linear cocycle $D_{\boldsymbol{x}}f_{\underline{\omega }}^n$ only sees the expanding direction associated to the top Lyapunov exponent. Proposition 4.1 can be regarded as a consequence of a random version of Oseledec’s Multiplicative Ergodic Theorem introduced by Kifer, that can be found in [32, Theorem III.1.2]. With these observation in mind, we find the following rather direct corollary.

### Corollary 4.2

Under the assumptions of Proposition 4.1 and hypotheses (H1)-(H3), if $\lambda _1>0$, then for any $\varepsilon \in (0,\lambda _1)$ and any $\boldsymbol{v}\in \mathbb {S}^2$, there exists $\hat{k}_1 = \hat{k}_1(\boldsymbol{x},\underline{\omega },\boldsymbol{v},\varepsilon )$ that is positive for a.e. $\boldsymbol{x}\in \mathbb {T}^3$ and $\mathbb {P}-$a.a. $\underline{\omega }\in \Omega$, such that

$$ |D_{\boldsymbol{x}} f_{\underline{\omega }}^n\boldsymbol{v}| \ge \hat{k}_1 \textrm{e}^{(\lambda _1-\varepsilon )n}, $$

holds true for all $n\in \mathbb {N}$, a.e. $\boldsymbol{x}\in \mathbb {T}^3$ and $\mathbb {P}-$a.a. $\underline{\omega }\in \Omega$.

### Proof

A direct application of Proposition 4.1 yields that for any $\boldsymbol{v}\in \mathbb {S}^2$, and for any $\varepsilon \in (0,\lambda _1)$, there exists $N\in \mathbb {N}$ for which

$$ \left| \frac{1}{n}\log |D_{\boldsymbol{x}} f_{\underline{\omega }}^n\boldsymbol{v}| - \lambda _1\right| <\varepsilon $$

holds true for a.e. $\boldsymbol{x}\in \mathbb {T}^3$, $\mathbb {P}-$a.a. $\underline{\omega }\in \Omega$, and every n≥N. In particular, we obtain that $\log |D_{\boldsymbol{x}} f_{\underline{\omega }}^n{\boldsymbol{v}}| > n(\lambda _1-\varepsilon )$, or equivalently

$$ |D_{\boldsymbol{x}} f_{\underline{\omega }}^n\boldsymbol{v}| > \textrm{e}^{(\lambda _1-\varepsilon )n}, $$

for a.e. $\boldsymbol{x}\in \mathbb {T}^3$, $\mathbb {P}-$a.a. $\underline{\omega }\in \Omega$, and every n≥N. To deal with n<N we use a classical result, see for instance [46], that states that for any matrix $M\in \mathbb {R}^{d\times d}$ and vector $\boldsymbol{w}\in \mathbb {R}^d$ there holds the bound

$$ |M\boldsymbol{w}| \ge (d-1)^{(d-1)/4}\frac{|\det M|}{\Vert M\Vert _F^{d-1}}|\boldsymbol{w}|, $$

where $\Vert \cdot \Vert _F$ denotes the Frobenius norm. In our specific setting, notice that we have $\det (D_{\boldsymbol{x}} f_{\underline{\omega }}^n)=1$ for all choices of parameters, and $\Vert D_{\boldsymbol{x}} f_{\omega }\Vert _F^2$ is bounded uniformly in $\underline{\omega }$ and $\boldsymbol{x}$, namely $\Vert D_{\boldsymbol{x}} f_{\omega }\Vert _F^2 \le K$ for some K≥1 that only depends on *U*, the upper bound of the parameters $|\textsf{A}|,|\textsf{B}|,|\textsf{C}|\le U$. Hence, for any fixed $\boldsymbol{v}\in \mathbb {S}^2$ we find that

$$ |D_{\boldsymbol{x}} f_{\underline{\omega }}^n\boldsymbol{v}| \ge \frac{\sqrt{2}}{K^n} $$

is satisfied for all values of $\underline{\omega }$ and $\boldsymbol{x}$. To conclude just notice that for all n≥N there holds $|D_{\boldsymbol{x}} f_{\underline{\omega }}^n\boldsymbol{v}| > \textrm{e}^{(\lambda _1-\varepsilon )n}$, while for all n<N we obtain $|D_{\boldsymbol{x}} f_{\underline{\omega }}^n\boldsymbol{v}| \ge \sqrt{2}K^{-N}$. Therefore there exists a constant $\hat{k}_1=\hat{k}_1(N)>0$ for which the claim of the corollary holds true. □

### Proof of Theorem 2

To prove Theorem 2 we need to compute the $L^1$ norm of the magnetic field $B(t,\boldsymbol{x})$ and apply Corollary 4.2. The growth of all the rest $L^p$ norms follows by Hölder’s inequality, since the domain is bounded. First of all, notice that because the vector field (1.8) is divergence-free, and hence the flow is volume preserving, there holds that

$$ \Vert B(t)\Vert _{L^1} = \int _{\mathbb {T}^3} |B(t,\boldsymbol{x})| \textrm{d}\boldsymbol{x}= \int _{\mathbb {T}^3} |B(t,\phi _t(\boldsymbol{x}))| \textrm{d}(\phi _t)_\#\boldsymbol{x}= \int _{\mathbb {T}^3} |B(t,\phi _t(\boldsymbol{x}))| \textrm{d}\boldsymbol{x}. $$

Then, using the convenient representation of *B* in terms of the flow (4.2),

$$ \Vert B(t)\Vert _{L^1} = \int _{\mathbb {T}^3} |D_{\boldsymbol{x}}\phi _t B_0(\boldsymbol{x})| \textrm{d}\boldsymbol{x}. $$

If we choose $t=n\in \mathbb {N}$, then the flow can be written as $\phi _t(\boldsymbol{x})=f_{\underline{\omega }}^n(\boldsymbol{x})$ and we are in position to apply Corollary 4.2 for discrete times $t=n\in \mathbb {N}$. Fix ε>0 and $B_0\in L^1(\mathbb {T}^3)$ with $\Vert B_0\Vert _{L^1}>0$. An application of Corollary 4.2 yields that there exists a (random) constant $\hat{k}_1$, that is positive for almost every $\boldsymbol{x}\in \mathbb {T}^3$, and $\mathbb {P}-$almost all $\underline{\omega }\in \Omega$, such that for all natural times $n\in \mathbb {N}$ we obtain

$$ {\begin{matrix} \Vert B(n)\Vert _{L^1} &  \ge \int _{\{\boldsymbol{x}\in \mathbb {T}^3 :|B_0(\boldsymbol{x})|\ne 0\}} \left| D_{\boldsymbol{x}}\phi _n \frac{B_0(\boldsymbol{x})}{|B_0(\boldsymbol{x})|}\right| |B_0(\boldsymbol{x})|\textrm{d}\boldsymbol{x}\\ &  \ge \textrm{e}^{(\lambda _1-\varepsilon )n} \int _{\{\boldsymbol{x}\in \mathbb {T}^3 :|B_0(\boldsymbol{x})|\ne 0\}} \hat{k}_1\left( \boldsymbol{x},\underline{\omega },\frac{B_0(\boldsymbol{x})}{|B_0(\boldsymbol{x})|},\varepsilon \right) |B_0(\boldsymbol{x})|\textrm{d}\boldsymbol{x}. \end{matrix}} $$

Since $\hat{k}_1$ is positive for almost every $\boldsymbol{x}$ and $\underline{\omega }$, and since we assume the set $\{\boldsymbol{x}\in \mathbb {T}^3:|B_0(\boldsymbol{x})|\ne 0\}$ to be of positive measure, then

$$ \hat{k}_2(\underline{\omega },B_0,\varepsilon ) = \int _{\{\boldsymbol{x}\in \mathbb {T}^3 :|B_0(\boldsymbol{x})|\ne 0\}} \hat{k}_1\left( \boldsymbol{x},\underline{\omega },\frac{B_0(\boldsymbol{x})}{|B_0(\boldsymbol{x})|},\varepsilon \right) |B_0(\boldsymbol{x})|\textrm{d}\boldsymbol{x}$$

is $\mathbb {P}-$almost surely positive.

Finally we can cover all times in between *n* and n+1 via a standard stability estimate. Let $a_\varepsilon (x) = (\varepsilon ^2+x^2)^{1/2}$, and define $|B|_\varepsilon = a_\varepsilon (B_1) + a_\varepsilon (B_2) + a_\varepsilon (B_3)$ so that $\int _{\mathbb {T}^3}|B|_\varepsilon \textrm{d}\boldsymbol{x}\rightarrow \Vert B\Vert _{L^1}$ and $a_\varepsilon '(x)\rightarrow \pm 1$ for all x≠0 as $\varepsilon \rightarrow 0$. Then we can write

$$ \frac{\textrm{d}}{\textrm{d}t}|B|_\varepsilon = \sum _{i=1}^3 a_\varepsilon '(B_i)\left( -(u\cdot \nabla )B_i + (B\cdot \nabla )u_i \right) = -(u\cdot \nabla )|B|_\varepsilon + \sum _{i=1}^3 a_\varepsilon '(B_i)(B\cdot \nabla )u_i, $$

and upon integrating and taking the limit $\varepsilon \rightarrow 0$, there yields that $\frac{\textrm{d}}{\textrm{d}t}\Vert B\Vert _{L^1}\ge -\Vert \nabla u\Vert _{L^\infty }\Vert B\Vert _{L^1}$. Hence, we obtain that $\Vert B(n+\tau )\Vert _{L^1} \ge \textrm{e}^{-\Vert \nabla u\Vert _{L^\infty }}\Vert B(n)\Vert _{L^1}$ for all $\tau \in (0,1)$. □

### Remark 4.3

Observe that the proof of ideal kinematic dynamo only requires a vector field with a flow satisfying Hypothesis (H1)-(H3), and with positive Lyapunov exponent and ergodic projective Markov chain. The ABC vector field (1.1) provides an example of such flow, but this is a general proof covering all vector fields with these properties.

## Data Availability

No datasets were generated or analysed during the current study.
